# Miniaturized Soft and Stretchable Multilayer Circuits through Laser‐Defined High Aspect‐Ratio Printing

**DOI:** 10.1002/smll.202501175

**Published:** 2025-05-27

**Authors:** Mohsen Mohammadi, Jin Shang, Yuyang Li, Aiman Rahmanudin, Darius Jakonis, Magnus Berggren, Lars Herlogsson, Klas Tybrandt

**Affiliations:** ^1^ Laboratory of Organic Electronics Department of Science and Technology Linköping University Norrköping 602 21 Sweden; ^2^ Wallenberg Wood Science Center ITN Linköping University Norrköping 602 21 Sweden; ^3^ Digital Systems Smart Hardware Printed, Bio‐ and Organic Electronics RISE Research Institutes of Sweden Södra Grytsgatan 4 Norrköping 602 33 Sweden

**Keywords:** printed electronics, soft electronics, stretchable electronics, wearables, wireless electronics

## Abstract

Stretchable electronics enable seamless integration of wearables with the human body, thereby creating new opportunities in biomedical applications. Miniaturized multilayer stretchable printed circuit boards are key for achieving high functional density circuits with minimal footprint. However, current microfabrication technologies struggle with simultaneously achieving tissue‐like softness (<<1 MPa), high resolution and low sheet resistance. This study demonstrates a scalable printing method that enables ultra‐soft (<0.4 MPa) stretchable conductors (>300% strain) with high‐resolution (<2.5 µm width) and high aspect‐ratio tracks (>1) connected by ultra‐fine (20 µm) vertical‐interconnect‐access (VIA) for multi‐layered configurations. The method is based on stencil printing into laser‐defined bio‐masks comprising the abundant biopolymer lignin, thereby achieving printing capabilities beyond conventional methods in a sustainable manner. Based on the unique capabilities, a miniaturized multilayer ultra‐soft wireless near‐field‐communication temperature logger is developed. Laser‐defined printing can pave the way for the next generation of ultra‐soft miniaturized wearables.

## Graphical abstract

A scalable printing method is developed that enables ultra‐soft stretchable conductors with high‐resolution and high aspect‐ratio tracks connected by ultra‐fine VIAs for multi‐layered configurations. The method uses stencil printing into laser‐defined bio‐masks comprising the abundant biopolymer lignin, thereby achieving printing capabilities beyond conventional methods in a sustainable manner. A miniaturized multilayer ultra‐soft wireless NFC temperature logger is developed as demonstration.

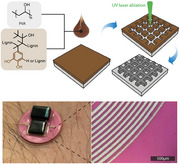

## 1 Introduction

Soft and stretchable electronics allows for seamless integration into deforming systems like clothing, skin, and tissue, thereby enabling next‐generation applications in soft robotics,^[^ ^1^, ^2^ ^]^ wearable electronics,^[^ ^3^, ^4^, ^5^ ^]^ health monitoring and therapeutics,^[^ ^6^, ^7^, ^8^ ^]^ and human‐machine interfaces.^[^ ^9^, ^10^ ^]^ Multilayered stretchable printed circuit boards (sPCB) is a key technology in enabling hybrid stretchable electronic circuits that can provide diverse functionality across a wide range of fields.^[^ ^11^, ^12^ ^]^ Achieving ultra‐soft (<<1 MPa) and miniaturized (<100 µm line width) devices is crucial for higher function density,^[^ ^12^, ^13^ ^]^ intimate coupling with biological tissues,^[^ ^14^ ^]^ reducing mechanical loads,^[^ ^15^ ^]^ and minimizing discomfort^[^ ^16^ ^]^ or immune responses.^[^ ^17^ ^]^ This requires ultra‐soft highly conductive and stretchable materials that can be efficiently printed in a scalable manner, ideally into high‐aspect ratio (AR) configurations that minimize footprint while maintaining low circuit resistance.

Several material approaches with distinct advantages and disadvantages have been explored for the realization of sPCBs, including structurally patterned metallic conductors,^[^ ^11^, ^12^, ^13^, ^18^ ^]^ liquid metals,^[^ ^19^, ^20^, ^21^, ^22^, ^23^ ^]^ and stretchable composites.^[^ ^24^, ^25^, ^26^, ^27^, ^28^ ^]^ Structural approaches employ conventional highly conductive metals (Au, Cu, Ag, Zn etc), but they undergo out‐of‐plane deformations that constrains resolution, softness, and conformability of these circuits. On the other hand, liquid metals are highly conductive and stretchable due to their fluidic nature,^[^ ^29^ ^]^ but due to alloying,^[^ ^30^ ^]^ robust interfacing with rigid circuit components is challenging. Stretchable composites based on a variety of metallic fillers with different shapes including nanoparticles,^[^ ^31^, ^32^, ^33^ ^]^ nanowires,^[^ ^28^, ^34^, ^35^ ^]^ and flakes^[^ ^24^, ^25^, ^36^, ^37^, ^38^, ^39^ ^]^ can be highly conductive and stretchable, but many of the high‐performing materials systems are rather stiff^[^ ^24^, ^25^, ^32^, ^33^, ^34^, ^35^, ^36^, ^37^ ^]^ and require complex processing,^[^ ^28^, ^32^, ^39^ ^]^ which is not consistent with high‐throughput printing processes. Therefore, to ensure practical applicability, the development of materials and processing methods that are efficient and scalable is required.

sPCBs employing stretchable composites have been established through various patterning methods, including photolithography,^[^ ^28^, ^34^, ^40^ ^]^ molding,^[^ ^41^, ^42^, ^43^ ^]^ laser ablation,^[^ ^44^ ^]^ photothermal patterning,^[^ ^25^ ^]^ 3D printing,^[^ ^26^, ^33^, ^45^, ^46^ ^]^ masked filtration,^[^ ^27^ ^]^ stencil printing,^[^ ^24^, ^36^, ^47^ ^]^ and screen printing.^[^ ^37^, ^48^ ^]^ Most of the reported sPCBs still have a high reported modulus (>1 MPa) beyond the range of human skin,^[^ ^24^, ^25^, ^26^, ^27^, ^28^, ^33^, ^34^, ^35^, ^36^, ^40^, ^41^, ^42^, ^44^, ^45^, ^47^, ^48^ ^]^ are not multi‐layered,^[^ ^33^, ^34^, ^37^, ^42^, ^43^, ^44^ ^]^ have a low AR^[^ ^24^, ^25^, ^27^, ^28^, ^34^, ^36^, ^37^, ^40^, ^44^, ^47^, ^48^ ^]^ (<<0.5) and low resolution^[^ ^25^, ^27^, ^41^, ^42^, ^43^, ^44^, ^46^, ^47^, ^48^ ^]^ (>100 µm). Stencil and screen‐printing methods allow for moderate resolution (down to 50 µm) and scalability, but they typically exhibit low ARs, especially at high resolutions. Although 3D printing offers high resolution (down to 3 µm) and high ARs (>1) prints, it is a serial printing process that has limited scalability, and the printing nozzles are easily clogged due to the aggregation tendency of the nanoparticles dispersions. While photolithography techniques offer high resolution, their AR is low (<<0.2) and they are not environment friendly due to use of unsustainable materials.^[^ ^49^, ^50^ ^]^ Furthermore, the use of harsh solvents and high‐temperature processes restrict their compatibility with most soft composite systems which restricts multilayer patterning. Photothermal patterning is scalable but limited in terms of resolution (down to 250 µm). Similarly, master moulding and direct laser ablation methods are highly capable of making high AR structures even for soft materials, but their resolution is often limited to 300 µm line widths and adapting them for scalable multilayered patterning is more demanding. In general, increasing the print resolution of stretchable conductors generally comes with a trade‐off in AR, sheet resistance, and stretchability. ARs and conductivity can be improved by increasing the filler density, but it normally results in a stiffer material system that limits softness and conformability. Therefore, the development of new printing methods for soft and stretchable conductors is needed to simultaneously achieve high‐resolution, high AR, and soft sPCBs.

Another important factor in the design of multilayered sPCBs is the implementation of stretchable conducting vertically interconnecting access (VIA) holes between the layers that are mechanically robust and scalable. VIAs have often been accomplished through direct writing,^[^ ^26^, ^45^ ^]^ patterned openings in insulating layers,^[^ ^27^, ^31^ ^]^ punching^[^ ^51^, ^52^, ^53^ ^]^ or drilling holes using laser ablation,^[^ ^12^, ^20^, ^22^, ^25^ ^]^ followed by filling of the hole with a conductive material. Creating VIAs in a scalable and robust manner for ultra‐soft miniaturized sPCBs is a major challenge due to the requirements for high‐resolution, alignment, and material selection. A related challenge is the robust mounting of rigid electronic components on sPCBs. This has often been achieved by the incorporation of stiffer^[^ ^24^, ^54^ ^]^ or rigid^[^ ^45^, ^55^ ^]^ layer/region to reduce the mechanical mismatch at the interface. Such approaches add complexity, stiffens the circuit, and can limit the resolution and density of the integrated components. Direct integration of rigid components into the soft sPCB^[^ ^25^ ^]^ is thus desirable but challenging as it often limits the stretchability of the circuit. In general, there are several fabrication challenges that needs to be addressed to achieve sPCBs that are ultra‐soft (<<1 MPa), multilayered with high resolution line width (<100 µm) and high AR (>0.5) conducting lines, and compatible with mounting of rigid components.

**Figure 1:**
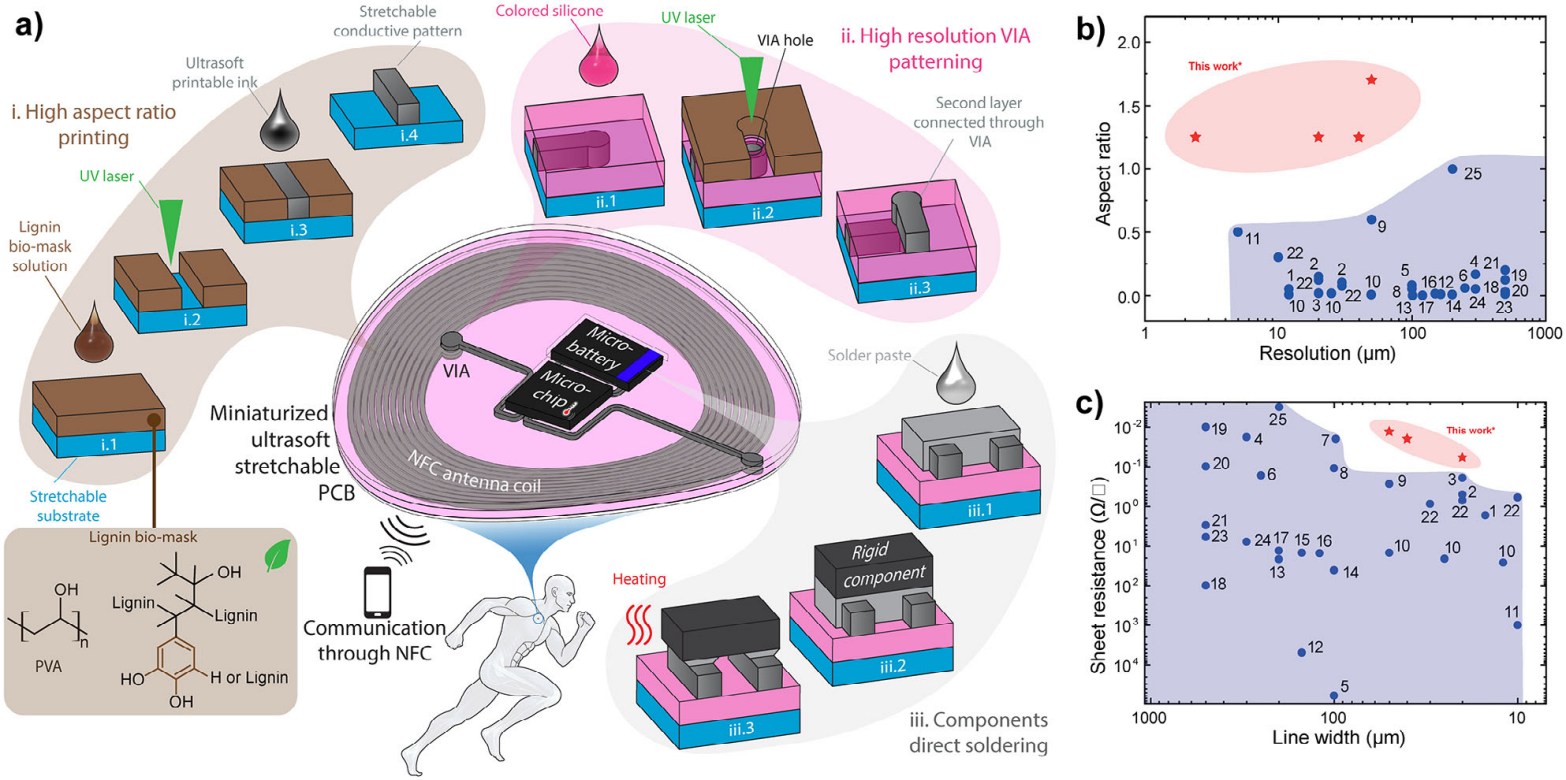
Laser‐defined stencil printing concept. a) Schematic of the developed method for printing of miniaturized ultrasoft stretchable PCBs. The fabrication steps include i.1 the coating of lignin stencil bio‐mask on a stretchable substrate, i.2 UV laser patterning of the bio‐mask, i.3 stencil printing of ultrasoft conductive ink in the bio‐mask and curing, i.4 dissolution of the bio‐mask in water. ii.1 coating of colored silicone on the first layer print, ii.2 UV laser patterning of stencil mask for the second layer and selective ablation of colored silicone for VIA formation, ii.3 stencil printing of second layer conductor. iii.1 stencil printing of solder paste, iii.2, placing circuit components, iii.3 direct soldering of components by heating in oven. b,c) Comparison of our printing method to previous reports of elastic stretchable conductors in terms of b) printing resolution and aspect ratio (see Table S1, Supporting Information for references) and c) the sheet resistance at different resolutions (see Table S1, Supporting Information for references).

Here, we report on a printing method based on high‐resolution laser‐defined sacrificial masks, made from a sustainable lignin‐based bio‐blend, for stencil printing of the ultra‐soft and stretchable silver flakes (AgFs) and elastomer composite conductive inks (**Figure** **1**a). The use of lignin addresses the sustainability challenges facing current microfabrication technologies where petroleum‐based polymers are typically used.^[^ ^56^, ^57^ ^]^ Briefly, the process starts with coating stencil bio‐mask on a stretchable substrate followed by high‐resolution laser patterning (down to 2.5 µm) of high AR (>1) openings into which the ultra‐soft conductive ink is stencil printed (Figure 1a,i). The method achieved ultra‐soft low resistance conductive tracks (<0.1 Ω/□) with linewidth down to 20 µm on elastomer substrates while using sustainable materials and mild processes. This has not been possible with previous techniques that generally achieves resistive tracks with ARs well below 1, resolutions above 10 µm, linewidths above 20 µm (see Figure 1b,c for the comparison with state‐of‐the‐art microfabrication techniques for elastic stretchable conductors). Our method can also achieve multilayered circuits by printing a second layer on top of a coated colored passivation layer, and at the same time form high resolution (20 µm) VIAs (Figure 1a,ii). Finally, rigid components can be directly soldered onto the sPCBs to create soft hybrid electronic circuits (Figure 1a,iii). To demonstrate the feasibility of the method for a wearable application, stretchable LED matrix displays and miniaturized wireless ultra‐soft wearable body temperature sensors comprising near‐field communication (NFC) and microbattery for mobile phone readout were developed.

## 2 Results and Discussion

### 2.1 Ultra‐Soft Printed Stretchable Conductors

**Figure 2:**
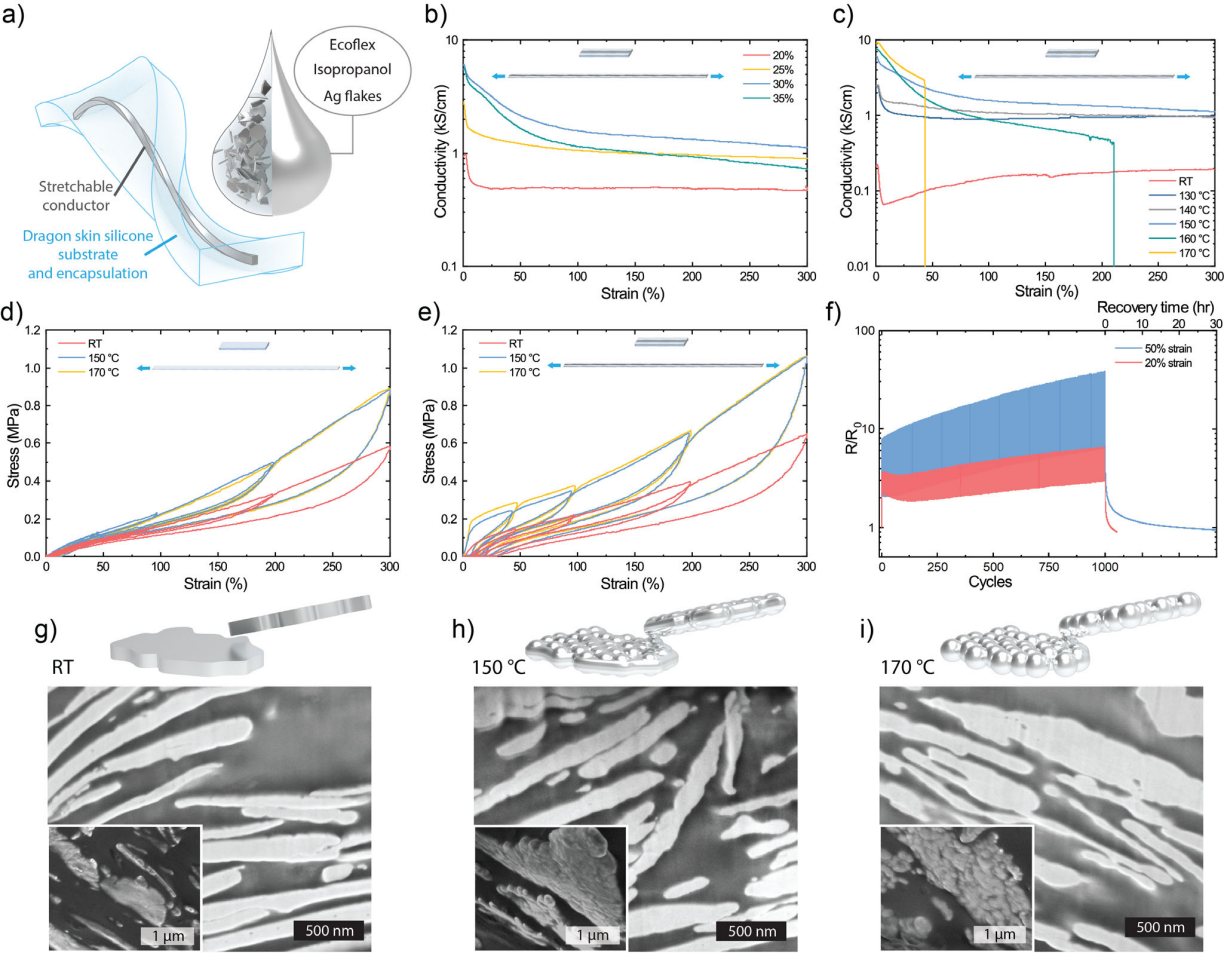
Printed stretchable conductor. a) Schematic of the stretchable conductor and ink composition. b) Conductivity vs strain for stretchable conductors with different loading of silver flakes (AgFs) sintered at 150 °C for 1 h. c) Conductivity vs strain for conductors with 30% AgFs loading sintered at different temperatures for 1 h. d) Stress–strain curves of Dragon skin (DS) silicone cured at different temperatures. e) Stress–strain curves of stretchable conductors on DS substrates sintered at different temperatures. f) Cyclic stability of stretchable conductor during 1000 strain cycles and recovery. g–i) Schematics of AgFs and SEM images of focused ion beam (FIB) milled samples. Insets show SEM images of liquid N_2_ cracked samples sintered at g) RT, h) 150 °C, and i) 170 °C.

To achieve printing of ultra‐soft sPCBs both material selection and processing methods need to be considered. The ultra‐soft (0.22 MPa) silicone elastomer Dragon Skin (DS) was selected as the substrate and encapsulation material (**Figure** **2**a) as its thermal and chemical stability ensures compatibility with the fabrication process. The addition of conducting fillers typically stiffens the composite, hence the even softer silicone rubber formulation Ecoflex 00–20 with a modulus of <0.06 MPa at 100% strain and maximum strain of >600%^[^ ^58^ ^]^ was chosen as the composite matrix. Ag flakes (AgFs) were selected as the conductive filler to simultaneously achieve high conductivity and printability. The stretchable conductive ink was prepared by mixing AgFs and Ecoflex 00–20 in isopropanol to produce a well dispersed ink of suitable viscosity for printing. To enhance the electromechanical performance of the composite, the loading of AgFs and the sintering process were optimized. The initial conductivity for 20, 25, 30, and 35 v% AgFs were 0.789 ± 0.147, 2.904 ± 0.233, 5.64 ± 0.182 and 5.33 ± 0.114 kS cm^−1^ (*n* =  3), respectively, with a sintering temperature of 150 °C (Figure 2b). The 30 v% of AgF composite demonstrated the best electromechanical performance among the tested formulations and maintained a conductivity of 1.1 kS cm^−1^ even at 300% strain. The electrical percolation network improves with filler loading, while the mechanical properties deteriorated at higher loadings of 35 v%, resulting in an optimum loading of 30 v% that has simultaneously good electrical and mechanical properties.

Various mechanisms can be employed for sintering AgFs to improve their electromechanical properties, including photo‐sintering,^[^ ^25^, ^59^ ^]^solvent‐sintering,^[^ ^60^ ^]^ mechanical deformation treatment,^[^ ^61^ ^]^ and thermal sintering.^[^ ^25^, ^36^, ^37^ ^]^ Here, we chose thermal sintering due to its effectiveness, scalability, and simplicity. The initial conductivity increases from 0.191 ± 0.018 to 8.66 ± 1.11 kS cm^−1^ (*n* =  3) for a sintering temperature of 170 °C (Figure 2c). An optimum sintering temperature was achieved at 150 °C, for which the conductor has the best electromechanical performance over a 300% strain range. At different curing temperatures of RT, 150 and 170 °C, the DS elastomer obtained a 100% modulus of 0.154 ± 0.004, 0.220 ± 0.009, and 0.214 ± 0.017 MPa (*n* =  3), respectively (Figure 2d). For printed composites with AgF, a similar effect in their 100% modulus with respect to curing temperature (RT, 150 °C, and 170 °C) was observed with a modulus of 0.195 ± 0.022, 0.372 ± 0.033, and 0.374 ± 0.004 MPa (*n* =  3), respectively (Figure 2e). The simultaneous embedment of softness and electrical performance into the composites makes it an ideal conductor for seamless integration with the human skin (Young's modulus ≤0.85 MPa)^[^ ^62^ ^]^. The conductor shows quite stable performance during 1000 stretching cycles at 20 and 50% strain (Figure 2f). Interestingly, their resistance recovered when the composites were in their relaxed state over time, eventually reaching below their initial values. This indicates that the composite has a viscoelastic behavior which allows it to flow and relax at long time scales.

The effect of the sintering of the AgFs in the composite were then investigated by cross‐sectional SEM (Figure 2g‐I; Figure S1, Supporting Information). Sintering at higher temperatures has several pronounced effects on the AgF that include the adoption of a rougher texture, welding of the flakes, and the formation of Ag particles between the flakes (Figure S3, Supporting Information). At 150 °C, the connectivity between the flakes provided high electrical conductivity while preserving good mechanical properties. At 170 °C, the flakes adopt an even rougher texture and they become even more connected, resulting in a slightly higher initial conductivity. However, at this point an extended flake network likely rigidifies the composite, making it vulnerable to fracturing even at moderate strains, as indicated by the backlight microscope images (Figure S2, Supporting Information) and deteriorating electromechanical performance (Figure 2c).

For the optimized conductor, no visible cracking was observed under backlight imaging (Figure S2, Supporting Information), and no delamination occurred throughout the electromechanical characterization, confirming the structural integrity of the printed traces. While some conductivity loss was observed during strain cycling (Figure 2f), this degradation was reversible, as conductivity consistently recovered upon relaxation. This indicates that the failure mechanism is likely due to reversible microscopic effects rather than macroscopic permanent structural damage. We speculate that the temporary conductivity loss is caused by microscopic rearrangements within the conductive network, such as slight separations of the flakes, which recover upon release of the mechanical load.

### 2.2 Laser Patterning of Sacrificial Stencil Masks

**Figure 3:**
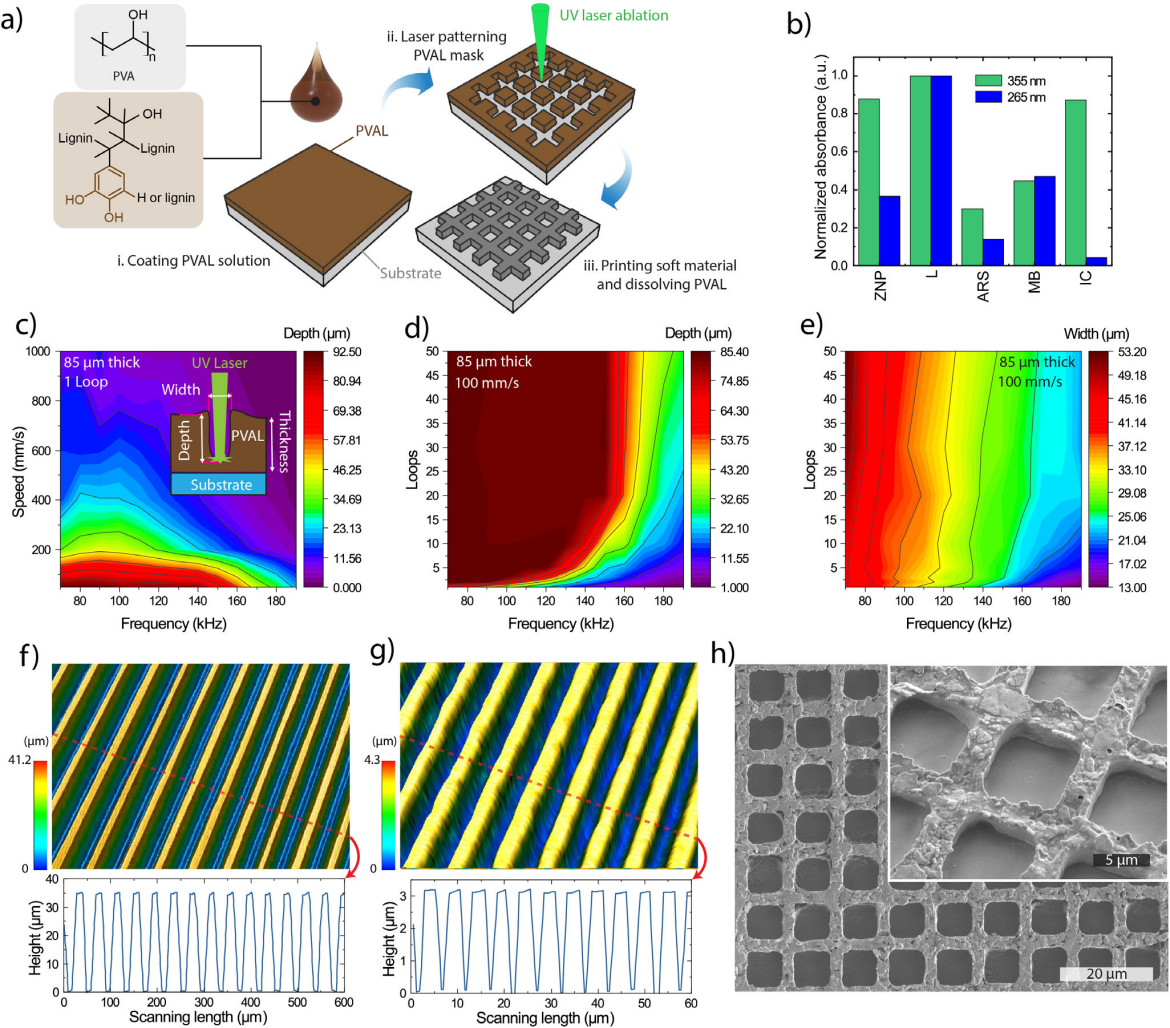
Laser defined sacrificial stencil mask. a) Schematic of the high‐resolution printing process with laser ablation defined bio‐blend mask. b) Absorbance of different polyvinyl alcohol (PVA) colorants including zinc oxide nano particles (ZNP), lignin sulfonate (L), Alizarin red S (ARS), methyl blue (MB), and indigo carmine (IC) at the two wavelengths of the lasers. c) Depth of the laser ablation of the PVAL at different frequencies and scanning speeds for the 355 nm laser (one loop). d) Depth and e) width of the laser ablation at different frequencies and loop counts at 100 mm s^−1^ speed. High resolution and high AR defined masks using f) 355 nm and g) 266 nm lasers. h) Printed stretchable conductor ink using a high resolution and high AR defined mask.

A major challenge associated with printing of soft high resolution and high aspect ratio structures is that the ink spreads out during and after the printing. To circumvent this issue, we developed a printing method based on a laser patterned sacrificial mask into which the ink was stencil printed (**Figure** **3**a). The approach has three main advantages including the use of a low viscosity ink while filling the high AR patterns without flowing out, the ink can be cured within the sacrificial mask that allows for printing of conventional silicone‐based composites, and high‐resolution patterns can be rapidly generated by laser ablation with a galvo scanner. Polyvinyl alcohol (PVA) and lignin sulfonate (L) were chosen as the base material for the mask as they are biodegradable^[^ ^63^, ^64^ ^]^ and can be processed in water, thereby removing the need for potentially harmful or harsh organic solvents (see Figure 3a for their molecular structure). As PVA has a low absorption in the UV spectrum, a variety of water soluble/dispersible colorants were evaluated to improve the UV absorption of the mask during laser exposure, and LS showed the highest absorption for both laser wavelengths (Figure 3b). Apart from absorption, the solubility of the ablated regions in water was also critical. Some of the colorants such as zinc oxide nanoparticles (ZNPs) and methylene blue (MB) resulted in insoluble material after laser ablation. For other dyes like Alizarin red S (ARS) and indigo carmine (IC), their solubility constituted a severe limitation. Additionally, lignin sulfonate is a sustainable biopolymer and it fulfils all processing requirements including the role of a thickening agent which can facilitate higher film thicknesses. Moreover, several layers of the PVA‐lignin sulfonate blend (PVAL) could be spin coated in sequence to reach higher thickness without any wrinkle formation.

The mask fabrication starts by coating PVAL onto an oxygen plasma activated substrate. Subsequently, a 355 nm or a 266 nm UV laser was used to ablate the features into the mask without damaging the underlying substrate due to the high UV absorption of PVAL. A 355 nm ns laser ablation system with galvo scanner was primarily used to rapidly (scan speed up to 10 000 mm s^−1^) ablate the mask. The laser had a fixed wavelength and pulse duration, and the tunable parameters that affected the quality of ablation of the masks were the pulse frequency (also affecting power, see Figure S4, Supporting Information), the scanning speed, and the loop count. As shown in Figure 3c and Figure S5 (Supporting Information), the depth and width of the ablation at a scanning speed of 100 mm s^−1^ enabled a wide range of different sizes while maintaining smooth ablation profiles (Figure S6, Supporting Information). To fine tune their properties, we explored the effect of the number of loops and pulse frequency on the width and depth of the patterns (Figure 3d,e; Figure S7, Supporting Information). For a fixed frequency, the number of loops does not substantially affect the width (Figure 3e), while the depth increases dramatically with increasing loop count (Figure 3d). Thus, the line width could be controlled by the pulse frequency (which affects the pulse power, see Figure S4, Supporting Information) while the loop count was used to achieve the desired thickness, although slightly higher resolution can be achieved for thinner masks (Figures S8 and S9, Supporting Information). The ablation results can be understood by considering that the laser beam has approximately a gaussian intensity profile in the radial direction. For lower pulse energies (higher frequency), the area above the ablation threshold is smaller, which makes the ablated spot smaller. Lower energy pulses remove less material, thus more loops are needed to reach a certain ablation depth. The final aspect ratio of an ablated line depends mainly on the laser beam shape, which is a property of the laser system. When optimizing the parameters for the 355 nm laser, features down to 20 µm with AR of more than 1 were obtained in the mask (Figure 3f). For the 266 nm ns laser, resolutions down to 2.5 µm and AR of more than 1.5 was achieved (Figure 3h). Based on this, we show that the printing of the soft conductor inks into such high‐resolution masks allows the formation of conductors with line widths down to 2.5 µm and an AR of more than 1 (Figure 3h; Figure S10, Supporting Information).

### 2.3 Laser‐Masked Printed Stretchable Conductors

**Figure 4:**
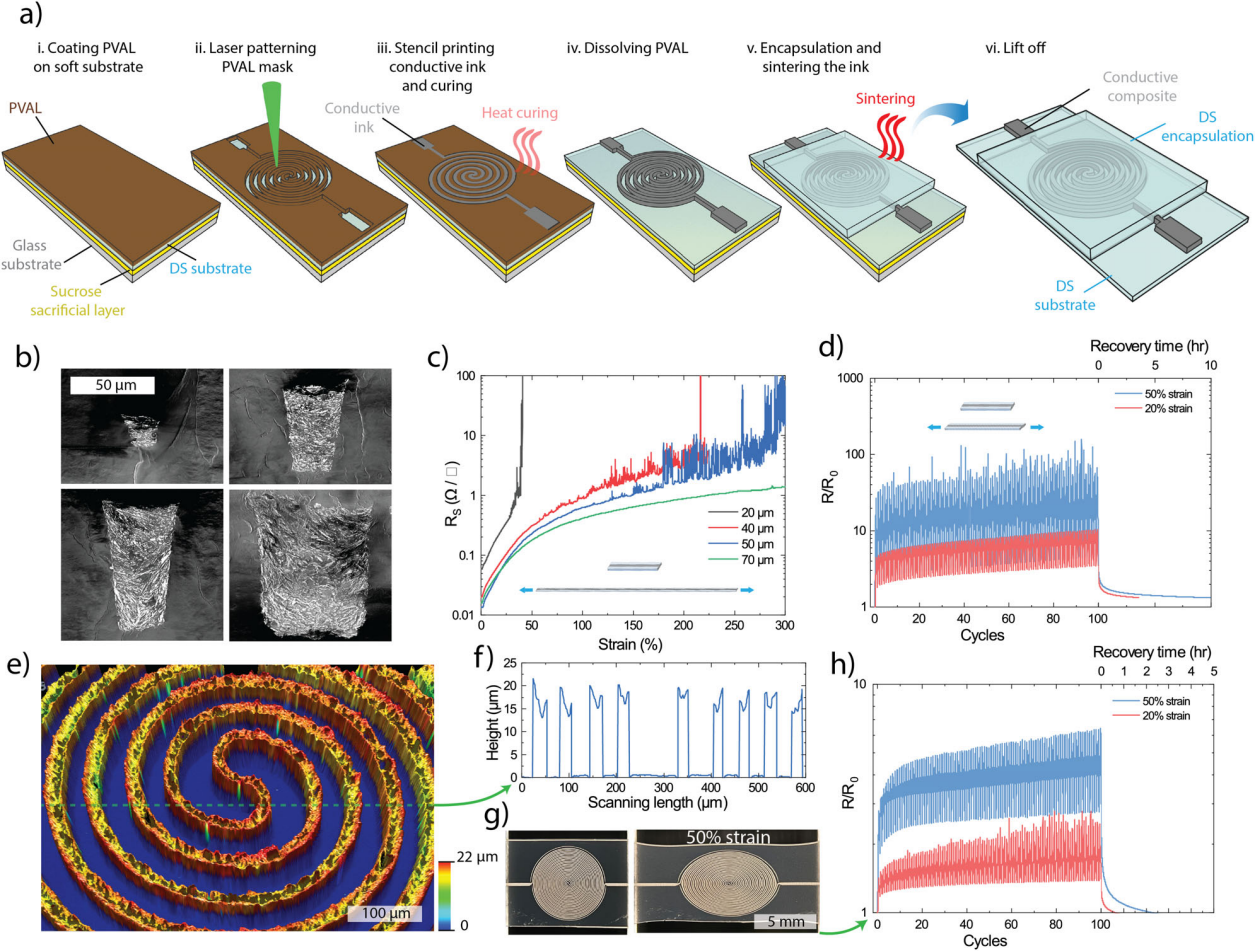
Laser‐masked printing of stretchable conductors. a) Schematic of the printing method for single layer printing of a stretchable conductor: i. Coating of stretchable substrate with PVAL layer. ii. 355 nm UV laser definition of the mask. iii. Stencil printing of the stretchable conductor and heat curing of the ink. iv. Dissolution of the sacrificial mask in water. v. Encapsulation with masked contacts and sintering of the composite conductors. vi. Release of the circuit by dissolving the sucrose layer in water. b) Cross section SEM images of different line widths and thickness of the print. c) Electromechanical performance of different conductor widths up to 300% strain. d) Strain cycling stability test of 70 µm line width conductor at 20% and 50% strains. e) 3D and f) 2D profiles of a 20 µm wide double spiral conductor print. g,h) 70 µm line width double spiral print g) image and h) strain cycling stability at 20% and 50% strains.

The processing steps to print a single layer ultra‐soft sPCB are shown in **Figure** **4**a. The lasered mask allows for arbitrary prints of high AR (Figure 4b), in contrast to conventional stencil printing through metal foil masks which has fundamental limitations in both print geometry and AR. The ink (Figure 2a) is printed into the mask using a soft squeegee to efficiently fill the mold and to remove any excess ink. The conductive print is then encapsulated with DS to improve the mechanical stability of the print. Despite achieving a 2.5 µm print resolution with this method (Figure 3g,h; Figure S10, Supporting Information), the particle size of the AgF is approximately between 2–5 µm. We therefore limited the study of the printed lines to widths equal to or more than 20 µm but smaller dimensions should be possible using smaller particles. The electromechanical performance of prints with widths of 20, 40, 50, and 70 µm were characterized by 4 probe measurements. The high AR is key for reaching ultralow sheet resistances down to 0.013 Ω/□ for high resolution prints and for maintaining the conductive percolation network during deformations (Figure 4c). In general, the sheet resistance of the conductive lines was below 0.1 Ω/□, and conductors with line widths of 20, 40 and 70 µm were able to retain stable conductivity up to 30%, 100% and 300% strain, respectively. The 70 µm lines showed good retention and recovery at 20% and 50% strain cycling (Figure 4d). To demonstrate high‐quality printing, high resolution and high AR, ultra‐long spiral prints were fabricated (Figure 4e,f). Optical images show a 25 cm long double spiral coil print of 70 µm width, which is more than 3500 squares in length (Figure 4g). The spiral patterns exhibited stable electromechanical performance and recovery over 100 strain cycles at 20% and 50% strain (Figure 4h), indicating excellent print quality with minimal defects.

Stretchable composite conductors typically show decreasing performance with decreasing line width, both with respect to conductivity and strain sensitivity.^[^ ^40^ ^]^ As the conduction relies on a finite number of particles and connections, by reducing the width the likelihood of losing the connective network due to local deformations or defects increase. Indeed, in our investigation (Figure 4c) we see a strong influence of conductor width on performance under strain. For 70 µm wide conductors, the sheet resistance increases smoothly with strain, while resistance noise appears at low strains for the narrower conductors. This can be understood as a changing network with few parallel pathways, in which the loss of one or a few critical connections can have a large influence on the total resistance. The earlier the noise appears, the earlier the conductor fails, which is consistent with the reasoning above. High aspect ratio structures help here, as a large cross section allows for more pathways and better stability. However, as the narrower conductors also are thinner due to printing limitations, the cross section decreases fast with width and thereby amplifies the strain sensitivity for narrow conductors. The performance of the narrow conductors should be possible to improve with a combination of high aspect ratio prints, smaller filler particle size and good particle dispersion. As the bending stiffness k_B_ ∝ Et^3^, where E is Young's modulus and t thickness, thicker prints need to be exceedingly softer to not induce substantial stiffening. Ultra‐soft conducting composites are thus key for realizing the benefits of high aspect ratio printing of stretchable electronics.

### 2.4 Hybrid Multilayer sPCBs

**Figure 5:**
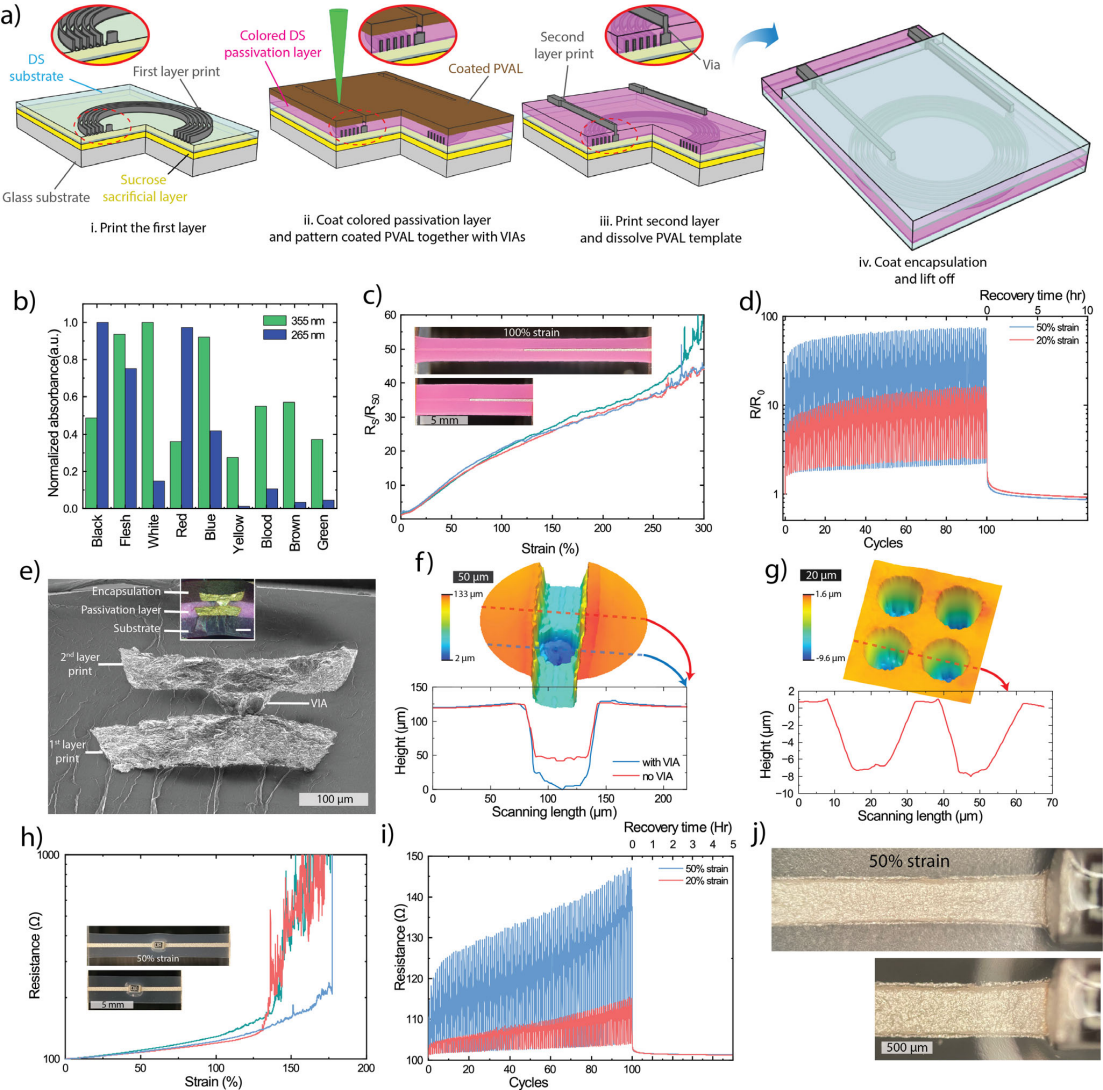
Multilayer hybrid sPCB fabrication. a) Schematic of the fabrication method: i. Printing of the first layer of conductor. ii. Coating of colored passivation layer and patterning of the second mask including VIAs. iii. Printing of the second layer and dissolution of the mask. iv. Coating of encapsulation and release of the device. b) Absorbance of various DS pigments at the two laser wavelengths. c) Electromechanical characterization and d) cyclic strain stability tests of two‐layer prints connected through a 70 µm in diameter VIA. e) SEM and optical microscope images of two printed layers connected through a 70 µm VIA. 3D and 2D profiles of laser drilled VIA using f) 355 nm and g) 266 nm lasers. h) Electromechanical, i) cyclic strain stability tests and j) optical microscopy of integrated rigid resistor directly soldered to the stretchable conductor.

The developed printing method is compatible with multilayer printing without addition complexity (**Figure** **5**a). After printing the first conductor layer, a colored passivation layer is coated as encapsulation. The enhanced UV absorption protects the conductor underneath from laser damage during subsequent patterning and allows for laser drilling of VIAs. Without colorant, the UV laser will pass through the DS layer and damage the conducting layer (Figure S11, Supporting Information). Figure 5b shows absorption of DS with different colorants. The concentration of colorant was tuned so that its ablation threshold was above that of PVAL, to enable PVAL patterning without damaging the passivation layer. We used 3% of the white colorant to improve the UV absorption in the film and 0.5% of the red colorant for visibility during fabrication. As the VIAs are formed in the second printing step, they require no additional processing and do not induce any mechanical mismatch within the structure. The formed VIAs were electromechanically stable and remain highly conductive even when stretched to more than 250% strain (Figure 5c). They also show stable electromechanical performance and recovery during cyclic strains up to 20% and 50% strain (Figure 5d). The method allows for stacking of additional layers, demonstrated by a three‐layer sPCB, with two interconnecting VIAs, which remained stable up to 200% strain (Figure S12, Supporting Information). Figure 5e displays SEM and optical microscopy images of the multilayer prints connected through a 70 µm VIA which was drilled through the colored passivation layer using the 355 nm UV laser. The resolution of the VIAs can be further improved if needed and reach down to ≈50 µm for the 355 nm laser and ≈ 20 µm for the 266 nm laser (Figure 5f,g).

A critical aspect for any sPCB technology is the mounting and electrical connection to rigid circuit components. Our approach is compatible with standard stencil printed low temperature solder to directly mount and connect surface‐mount devices (SMDs) onto our stretchable conductor. This enables conventional scalable pick‐and‐place integration of rigid components onto the stretchable circuit, making it compatible with traditional PCB processing. A 100 Ω resistor was used to investigate the stability of the connection to the SMD during stretching of the circuit. The connection was electromechanically stable up to 120% strain (Figure 5h) and during 100 strain cycling to 20% and 50% strain (Figure 5i). The results shows that the developed printing method for ultra‐soft sPCB technology is compatible with multilayer high‐resolution printing with the potential of designing sPCBs with high density SMD integration.

### 2.5 NFC Temperature Logger and LED Display

**Figure 6:**
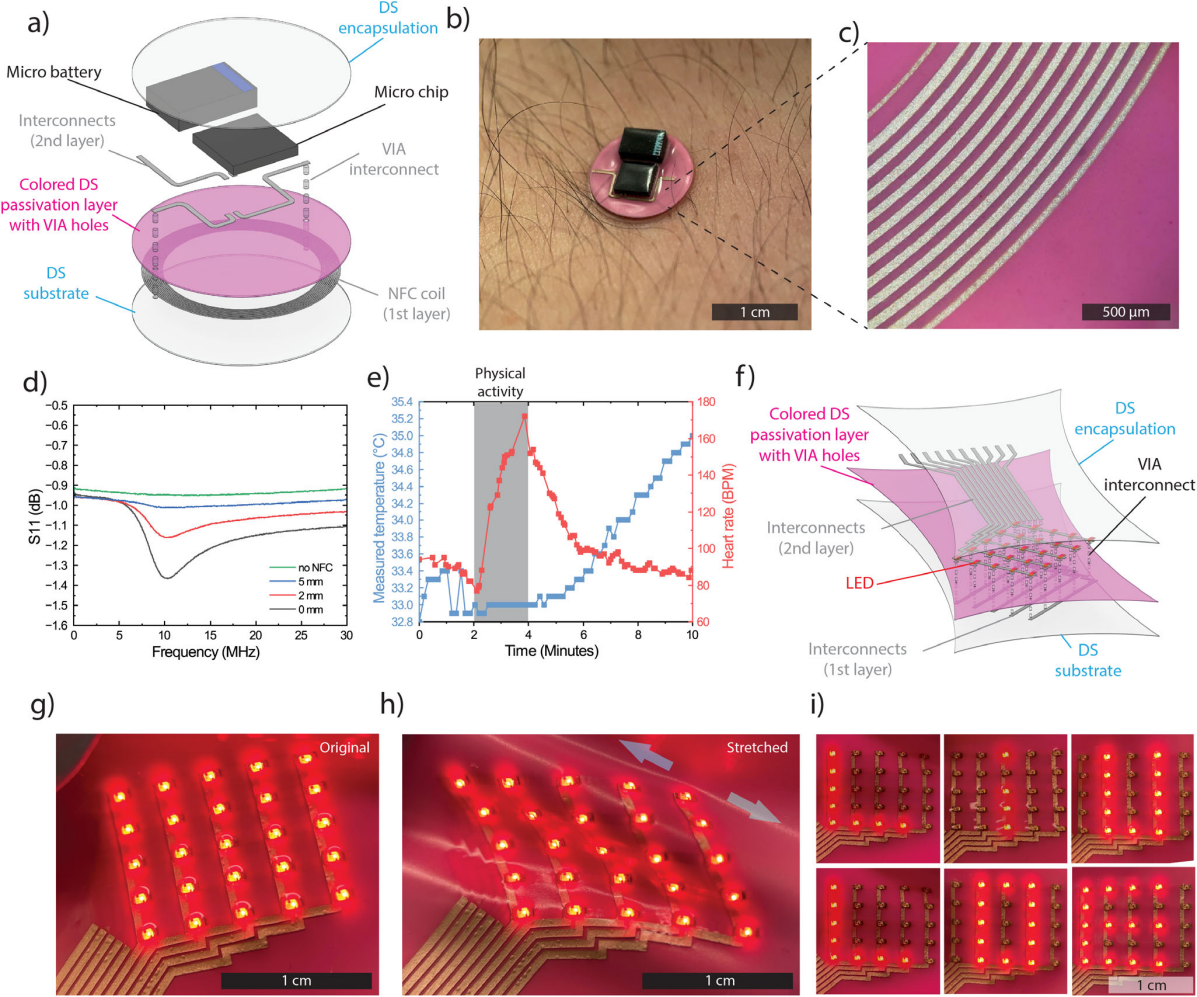
Ultra‐soft miniaturized NFC temperature logger and LED display. a) Schematic of soft temperature logger circuit comprising of a first layer with NFC antenna coil, a passivation layer with VIA holes, a second layer of interconnects, integrated microchip and micro battery, and encapsulation. b) A soft miniaturized temperature logger device on a fingertip. c) Microscope image of the embedded high‐resolution NFC coil taken from the backside of the device. d) S11 Parameter of the miniature NFC antenna as a function of frequency at various distances from the reader. e) Body temperature change measured by the soft temperature logger before, during, and after a physical activity (heart rate also shown). f) Schematic of a printed LED matrix display, including a first layer of printed conductors, a passivation layer with VIA holes, a second layer of printed conductors, integrated LEDs, and encapsulation. g) A stretchable LED matrix display in relaxed and h) stretched state. i) Demonstration of the addressability of the LED matrix.

The high resolution and excellent performance of the developed sPCB technology was leveraged to develop an ultra‐soft miniaturized wireless temperature logger (11 mm in diameter). The circuit comprises a first layer of the NFC antenna coil on DS substrate, colored DS passivation layer with VIA holes, a second layer of interconnects, a sensor‐integrated microchip, a micro‐battery, and lastly an encapsulation layer (**Figure** **6**a). The device was capable of recording temperature through the integrated NFC chip (Figure 6b) over an extended period with the integrated micro battery (characterized in Figure S13, Supporting Information). The recorded data could be wirelessly transmitted to a smartphone through the soft miniaturized NFC antenna, which was made up of a 32 cm long and 70 µm wide stretchable coil (Figure 6c). The coil was designed to communicate with external readers by magnetic inductive coupling at a resonance frequency ≈13.56 MHz (Figure 5d). The device allowed for a wireless readout up to 3 mm in distance and the signal declined with increasing distance as expected for NFC (Figure 6d). The soft device was used to record skin temperature before, during, and after physical activity. Following each trial, the data was wirelessly transmitted to a smartphone. The device was tested in three trials, performing successfully in all instances. Variations in the temperature response were observed, likely due to differences in the specific experimental conditions (Figure 6e; Figure S14, Supporting Information). Our ink and printing method is also compatible with higher frequency antennas, demonstrated by the printing and characterization of an RFID antenna with resonance frequency within the RFID range of the EU and the US (0.863 GHz to 0.928 GHz) (Figure S15, Supporting Information).

To further demonstrate the facile integration of SMDs onto the multilayer sPCBs, a 5 × 5 LED matrix display was designed and fabricated (Figure 6f). The device was made by printing a first layer of conductors on a DS substrate, a passivation layer with 30 VIA holes, a second layer of printed conductors, stencil printed solder paste with 25 soldered LEDs, and an encapsulation. Low‐light red LEDs (630 nm) have been shown to be effective to improve skin regeneration^[^ ^65^ ^]^ and were therefore chosen for this demonstrator. The soft display is conformable and stretchable (Figure 6g,h), and each LED can be controlled individually with an Arduino Uno controller (Figure 6i).

## 3 Conclusion

We have demonstrated a novel printing method for achieving high resolution and high AR conductors for ultrasoft and stretchable multilayer sPCBs. The key innovation is the development of direct and selective laser patterned sacrificial bio‐masks on ultra‐soft elastomer substrates followed by the stencil printing of high resolution ultra‐soft conductive prints reaching as fine as 2.5 µm line widths. The use of the biopolymer lignin in sacrificial bio‐masks that can be processed with aqueous solvents provides a pathway for sustainable microfabrication technologies for soft electronic systems. Additionally, we developed an ultra‐soft stretchable conductor with exceptional electromechanical performance, characterized by high conductivity (5640 S cm^−1^), stretchability (>300% strain), and the capability for miniaturization down to 20 µm line widths enabled by our stencil bio‐mask method. Multilayer printing of the circuits has been made possible by the development of a colored passivation layer, which serves to protect the bottom layer from laser‐induced damage and enables high resolution VIA formation. Rigid circuit components have been seamlessly integrated into the stretchable sPCBs using a direct soldering method. We also demonstrated the feasibility of our method by fabricating a stretchable LED matrix emitting deep red light that has the potential for therapeutic applications, and a microbattery powered miniaturized ultra‐soft temperature logger capable of monitoring real‐time physiological parameters and transmitting data wirelessly to a smartphone through an integrated stretchable NFC antenna. We believe that this printing technology possesses significant capabilities for scalable production of intricate stretchable and ultra‐soft devices with high‐density component integration suitable for a variety of next‐generation e‐wearables and e‐implants.

## 4 Experimental Section

### Substrate Preparation

Glass slides were cleaned using a UV ozone cleaner (PSD‐UV8, Novascan) for 15 min before spin coating 10%wt solution of sucrose (Sigma–Aldrich, 99.5%) in DI water at 2000 rpm for 30 s, followed by drying on a hotplate at 70 °C for 5 min. The sacrificial layer was selectively removed from the edges of the glass slides using a UV 355 nm pulsed ns laser engraver (3 W, FMHUV3W, MetaQuip Laser engraving machine) (1 loop, frequency 30 kHz, 100 mm s^−1^ speed) to seal the sacrificial layer from dissolving in DI water during the processing. The substrate was then treated with an oxygen plasma cleaner (Zepto W6 Plasma Cleaner, Diener) at 50 W for 2 min to enhance the adhesion of the subsequent layer. Next, Dragon Skin 10 slow (Smooth‐On, 1:1) was mixed using a planetary centrifugal mixer (ARE‐250, Thinky) at 2000 rpm for 1 min, followed by degassing through centrifugation at 2200 rpm for 30 s. Dragon Skin 10 slow was spin coated at 2000 rpm for 30 s to form a 130 µm thick, soft substrate layer. Finally, the substrate was placed on a hotplate at 70 °C for 10 min for curing.

### Ink Preparation

Two‐component Ecoflex 00–20 (Smooth‐On, 1:1) was mixed as above, and then isopropanol (VWR, 98%) was added, and the solution was mixed again at same conditions. Ag flakes powder (47MR‐11F, Inframat Advanced Materials, 99.95%, average particle size: 2–5 µm) was added to the solution and the ink was mixed five times at the same conditions. The weight ratio of the ink components was consistently 1.43:1:6 for Ecoflex:isopropanol:AgF (for reaching 30%v of AgF) unless otherwise stated. The IPA volumetric ratio in the ink was consistently maintained at 40%v.

### Stencil Printing of Test Samples

A 70 µm thick plastic mask was placed on the stretchable substrate and the ink was stencil printed. Subsequently, the mask was removed, and the sample was cured on a hotplate at 60 °C for 1 h to evaporate the solvent, followed by increasing the temperature to 80 °C and maintaining for 30 min to cure the ink. The contact pads were covered with 130 µm thick DS strip masks. The sample was spin coated with dragon skin 10 slow premixed solution at 2000 rpm for 30 s. The DS mask was removed, and the sample was placed in a vacuum desiccator for 2 min to remove any trapped air bubbles. The sample was then cured at 70 °C for 10 min on a hot plate to form the encapsulation and sintered at 150 °C for 1 h in an oven, unless otherwise stated. Stencil printed conductors were 500 µm wide, 15 mm long, and ≈60 µm thick.

### Bio‐Composite Mask Preparation

PVA (Mowiol 18–88, Sigma–Aldrich) was dissolved by stirring in DI water in a water bath at 80 °C overnight to achieve a concentration of 20%wt. The colorant (ZnO NPs (nanopowder, <50 nm particle size, Sigma–Aldrich, 97%), Methyl blue (Sigma–Aldrich), Indigo carmine (Sigma–Aldrich), Alizarin red S (Sigma–Aldrich), or Lignin (average Mw 7k, 3% of sulfonic acid group, supplied by Domsjö)) was added to the solution to reach 10%wt in the final film. Excess DI water was added to reach a final concentration of 16%wt PVA in DI water. The solution was stirred in a water bath at 80 °C overnight and allowed to cool down under ambient conditions. To enhance the adhesion of the bio‐composite to the substrate, the substrates were treated with oxygen plasma at 50 W for 5 min before spin coating the bio‐composite solution. The solution was spin coated and placed on a hot plate at 70 °C to evaporate the water. To achieve various thicknesses, the solution was spin‐coated at different speeds, allowing for thicknesses up to 50 µm. For higher thicknesses, the layer was spin‐coated atop another dried layer. Layers thinner than 10 µm were spin‐coated using a diluted solution (8%wt).

### Laser Ablation of Masks

Two pulsed nanosecond UV laser engravers were used to ablate the masks without damaging the underlying layers: a UV 355 nm laser (FMHUV3W, Poplar‐355‐3A5, MetaQuip Laser Engraving Machine; 3 W, 20 ns pulse width) and a UV 266 nm laser (MicroMake Plus 266, Bright System s.r.l.; 50 mW). The output power and pulse energy of the 355 nm laser at different frequencies were measured using a power/energy meter (Model 841‐PE, Newport), as shown in Figure S4 (Supporting Information). The laser parameters were adjusted to define distinct patterns on masks of varying thicknesses. For resolution tests and creating the spiral samples, 20 µm width tracks were printed by one line with 35 µm thick masks (UV 355 nm laser, 20 loops, 100 mm s^−1^, 130 kHz). For 40 µm wide tracks, three 10 µm spaced parallel lines on 60 µm thick masks were used (UV 355 nm laser, 20 loops, 100 mm s^−1^, 130 kHz). For 50 µm width tracks, three 10 µm spaced parallel lines on 85 µm thick masks were employed (UV 355 nm laser, 30 loops, 100 mm s^−1^, 130 kHz). To achieve 70 µm width, five 10 µm spaced parallel lines on 85 µm thick masks were used (UV 355 nm laser, 30 loops, 100 mm s^−1^, 130 kHz). For parallel lines, the best profiles were achieved when the lines were scanned from the sides to the center, for example, for 5 parallel lines, this was the sequence: 15243. For high‐resolution masks, 3 µm thick masks were patterned by one line using a UV 266 nm pulsed ns laser engraver (60% power, 50 loops, 10 mm s^−1^, 55 kHz) to create 2.5µm wide features in the mold.

### Single Layer Printing

The UV 355 nm laser or UV 266 nm pulsed ns laser engraver was used to selectively ablate the Lignin‐PVA (LP) masks without altering the underlying layers. Before printing the conductive ink, the masks were cleaned by washing with isopropanol using a foam swab. The composite ink was stencil printed into the mask using a soft squeegee (folded 2.5 mm DS sheet, Figure S16, Supporting Information). After curing of the ink (60 °C 1 h, 80 °C 30 min on a hot plate), the sample was placed upside down in a water bath at 60 °C for 60 min to dissolve the sacrificial mask. After removing it from the bath, the sample was gently washed with ethanol (Solveco, 70%) to reduce surface tension during drying and prevent deformation of the high aspect ratio prints caused by water's high surface tension. The sample was placed on a hot plate at 60 °C for 10 min to dry. The contact pads were covered with 130 µm thick DS strip masks. The sample was spin coated with DS premixed solution at 2000 rpm for 30 s. The DS mask was removed, and the sample was placed in a vacuum desiccator for 2 min to remove any trapped air bubbles. Then the sample was cured at 70 °C for 10 min on a hot plate to form the encapsulation. The print was sintered at 150 °C for 60 min and released from the glass by laser cutting the edges (UV 355 nm laser, 20 loops, 100 mm s^−1^, 30kHz) and dissolving the sacrificial layer in DI water.

### Multilayer Fabrication

After printing the first layer, a colored passivation layer was directly coated on the print, instead of encapsulation. To make the passivation layer, white and red pigments (Silc‐Pig, Smooth‐On) was added to the part A component of DS and mixed, and then part B was added and the solution was mixed again. The weight ratio of the components was 100:3:0.5 for DS:white:red. The solution was spin coated at 2000 rpm for 60 s and cured at 70 °C for 10 min on a hot plate to form a 100 µm thick passivation layer. Then a mask layer was formed on the top of the oxygen plasma treated passivation layer and UV 355 nm laser (cross hatching mode, 1 loop, 100 mm s^−1^, 160kHz) was used to define the pattern in the second mask without damaging the passivation layer. Then VIA holes were drilled through the passivation layer (UV 355 nm laser, hatching mode, 3 loops, 100 mm s^−1^, 130 kHz). Then the second layer was stencil printed, cured, and encapsulated in the same manner as the first print. The samples were then sintered, released from the glass substrate, and used for characterization. Three‐layer conductor samples were fabricated using the same method, with the third layer printed by repeating the second‐layer fabrication process.

### Integration of Rigid Components

For the test samples, two conductive tracks that were separated by a 2 mm gap was stencil printed on the substrate. The ink was cured at 60 °C for 1 h and 80 °C for 30 min on a hot plate. Then the low temperature soldering paste (SSLTNC‐T5‐15G, SRA Soldering Products) was applied to the ends of a resistor (100 Ω, 0603, RND Electronics) and placed on the gap of the conductive tracks. The sample was placed in an oven at 150 °C for 5 min to solder the resistor to the conductive tracks. Then the sample was treated with oxygen plasma at 50 W for 5 min and coated with the DS premixed solution for encapsulation. The sample was placed in a vacuum desiccator for 2 min to remove the trapped air bubbles before placing on the hot plate at 70 °C for 10 min for curing. Finally, the samples were placed in an oven at 150 °C for 55 min for sintering the AgFs. Then the samples were released from the glass by laser cutting the edges and dissolving sacrificial layer in DI water.

### Temperature Logger Fabrication

The first layer comprising the NFC coil (10.5 turns, inner diameter 8.4 mm, outer diameter 10.6 mm, wire width 70 µm, wire spacing 30 µm) was printed on the substrate using a laser defined (UV 355 nm laser, cross hatching mode, 2 loops, 100 mm s^−1^, 160 kHz) 85 µm bio‐composite mask. The second layer was printed on the passivation layer with 200 µm wide and 50 µm thick conductor lines connecting a microchip (NHS 3100, NXP Semiconductors) to a 100 µAh micro battery (ITX181210AABBAA, Iten) and the NFC through two 300 µm VIAs (UV 355 nm laser, cross hatching mode, 2 loops, 100 mm s^−1^, 130 kHz) using a 60 µm thick bio‐composite mask (UV 355 nm laser, cross hatching mode, 1 loops, 100 mm s^−1^, 160 kHz). The sPCB was sintered at 150 °C for 25 min prior to integration of the rigid components. The solder paste was stencil printed on the stretchable conductor print using a 355 nm UV laser defined 25 µm thick plastic mask. The rigid components were placed on the solder paste print and baked at 150 °C for 3 min. After the oxygen plasma treatment of the sample, the device was encapsulated by a 130 µm DS and cured at 70 °C for 10 min and at 150 °C for 2 min. The micro battery was charged using a potentiostat (1001E, Gamry). The device was transferred onto a thin 50 µm Ecoflex gel substrate to enhance skin adhesion. It was placed on the upper chest of the study participants and gently pressed to ensure proper contact. The subjects sat for 3 min, ascended a flight of stairs for 2 min, and then sat again for an additional 6 min. Temperature was measured on the chest and read out from the device using a smartphone, while heart rate was simultaneously monitored with an Apple Watch. The experiments involving human subjects were approved by the Swedish Ethical Review Authority (Dnr. 2024‐06705‐01) and conducted in compliance with the ethical standards of Linköping University, as well as Swedish and European regulations. Informed written consent was obtained from all participants prior to the research.

### LED Matrix Display Fabrication

The first interconnect layer was printed on the prepared substrate using a laser defined (UV 355 nm laser, cross hatching mode, 2 loops, 100 mm s^−1^, 130 kHz) 85 µm bio‐composite mask. The second interconnect layer including VIAs and contacts to LEDs (632 nm, SML‐P11VTT86R, ROHM Semiconductor) through 300 µm VIAs (UV 355 nm laser, cross hatching mode, 2 loops, 100 mm s^−1^, 130 kHz) was printed on the passivation layer using an 85 µm thick bio‐composite mask (UV 355 nm laser, cross hatching mode, 2 loops, 100 mm s^−1^, 130 kHz). The sPCB was sintered at 150 °C for 25 min prior to integration of the LEDs. The solder paste was stencil printed on the stretchable conductor print using a 355nm UV laser defined 25 µm plastic mask. The LEDs were placed on the solder paste print and baked at 150 °C for 3 min. After the oxygen plasma treatment of the sample, the device was encapsulated by a 130 µm DS and cured at 70 °C for 10 min and at 150 °C for 2 min. The device was released from the glass slide by dissolution of sucrose sacrificial layer in water after laser cutting the edges.

### Characterization

Resistance‐strain measurements were performed using a motorized linear stage (X‐LSQ300A‐E01, Zaber) and 4 probe resistance measurements using a multimeter data acquisition system (Keithley 2701 Ethernet). Stress–strain measurements were performed using a custom‐made stress–strain setup consisting of a motorized linear stage (X‐LSQ300A‐E01, Zaber) coupled with a force gauge (M5‐012, Mark‐10), (conductor size: 500 µm wide and 50 µm thick conductor, sample size: 1.5 mm wide and 260 µm thick sample, length = 15 mm). The test samples were strained to 300% at 0.3 mm min^−1^ and cycled to 20% and 50% strain at 6 mm min^−1^. For the conductivity measurements, the thickness of the conductor samples was characterized by optical microscopy of the cross section of liquid N_2_ cracked samples. The length of resistance‐strain tests samples for high resolution and VIA characterization were 14.5 mm long. All scanning electron microscopy (SEM) imaging were done using a Sigma 500 Gemini (Zeiss). The effect of the sintering temperature on the AgFs were characterized by SEM imaging of liquid N_2_ cracked samples and the cross‐section was characterized by focused ion beam (FIB) milling (Zeiss cross beam 1540 ESB system, 5nA, 30kV). During the FIB milling, the sample was loaded on a tilted sample stage (54°). The ion‐milling procedure created trenches of 10 × 10 × 10 µm (length, depth, width). The optical absorption spectra measurements were obtained using Absorption Spectrometer Lambda 900 (Perkin Elmer Instruments). Optical surface profilometry of the samples were done using an optical 3D surface profiler (PLu neox 3D, Sensofar). Galvanostatic charge–discharge (GCD) curves were obtained using a potentiostat (1001E, Gamry). The reflection coefficient of the NFC antenna was measured by a network analyzer (Wimo, Mini VNA pro) with a primary coil over a frequency range of 1 to 30 MHz. The distance between the antenna and the primary coil was controlled by a height gage (Mitutoyo, Digimatic Height gage). The printed RFID antenna was designed based on a 0.25 λ meander dipole antenna^[^ ^66^ ^]^ and the S11 parameter of the antenna was measured by the Vector Network Analyzer (ZVA 24, Rohde&Schwarz).

## Conflict of Interest

The authors declare no conflict of interest.

## Supporting information



Supporting Information

## Data Availability

The data that support the findings of this study are available from the corresponding author upon reasonable request.
