# Supplementary material for: Miniaturized Soft and Stretchable Multilayer Circuits through Laser‐Defined High Aspect‐Ratio Printing
Source: Small. 2025 May 27;21(29):2501175. doi: 10.1002/smll.202501175 (PMC12288821; doi:10.1002/smll.202501175)
Supplement: Supplementary file 1 — Supporting Information [file SMLL-21-2501175-s001.docx]

**Supporting information**

**Miniaturized Soft and Stretchable Multilayer Circuits through Laser-Defined High Aspect-Ratio Printing**

*Mohsen Mohammadi,^1,2^ Jin Shang,^1,3^ Yuyang Li,^1^ Aiman Rahmanudin,^1,2^ Darius Jakonis,^3^ Magnus Berggren,^1,2^ Lars Herlogsson,^3^ and Klas Tybrandt^1,2^**

*^1^ Laboratory of Organic Electronics, Department of Science and Technology, Linköping University, 602 21 Norrköping, Sweden.*

*^2^ Wallenberg Wood Science Center, ITN, Linköping University, 602 21 Norrköping, Sweden.*

*^3^ RISE Research Institutes of Sweden, Digital Systems, Smart Hardware, Printed, Bio- and Organic Electronics, Södra Grytsgatan 4, 602 33 Norrköping, Sweden.*

*Email:* [*klas.tybrandt@liu.se*](mailto:klas.tybrandt@liu.se)

**Table S1** Comparison of the developed printing method to the state-of-the-art stretchable elastic conductors.

| **Ref** | **Printing**  **Method** | **Conductor/filler type** | **Scalable** | **Multi**  **layer** | **Line width [µm]** | **R_s_**  **[**Ω/□**]** | **Res.**  **[µm]** | **Thickness [µm]** | **Aspect Ratio** | **Modulus @100%**  **[MPa]** |
| --- | --- | --- | --- | --- | --- | --- | --- | --- | --- | --- |
| This work | Stencil printing | Ag Flakes | Yes | Yes | 20 | 0.059 | 2.4 | 3 | 1.25 | 0.37  (Dragon Skin) |
|  |  |  |  |  | 40 | 0.020 | 20 | 25 | 1.25 |  |
|  |  |  |  |  | 50 | 0.013 | 40 | 50 | 1.25 |  |
|  |  |  |  |  | 50 | 0.013 | 50 | 85 | 1.7 |  |
| ^1^ | Photo-lithography | AgNWs | No | Yes | 15 | 1.700 | 12 | 0.59 | 0.049 | >1 (PDMS) |
| ^2^ | Photo-lithography | AuNWs | No | No | 20 | 0.500 | 20 | 3 | 0.15 | >1 (PDMS) |
| ^2^ | Photo-lithography | AuNWs | No | No | 30 | 0.544 | 30 | 3 | 0.10 | >1 (PDMS) |
| ^3^ | Photo-lithography | AgNWs and AuNWs | No | Yes | 20 | 0.192 | 20 | 0.325 | 0.016 | >1 (SEBS H1041) |
| ^4^ | Moulding | AgNWs | Yes | No | 300 | 0.017 | - | 50 | 0.17 | >1 (SBS) |
| ^5^ | Laser ablation | Carbon | Yes | No | 100 | 6 x 10^4^ | 100 | 8 | 0.08 | >1 (PDMS) |
| ^6^ | Photothermal lithography | Ag flake | Yes | Yes | 250 | 0.167 | 250 | 14 | 0.056 | 2 (PDMS) |
| ^7^ | 3D printing | Ag particles and MCNTs | No | Yes | 98 | 0.020 | 86 | N.R | N.R | >1 (PDMS) |
| ^8^ | Masked filtration | AgNWs | No | Yes | 100 | 0.110 | 100 | 5 | 0.05 | >1 (PDMS) |
| ^9^ | Stencil printing | Ag flake | Yes | Yes | 50 | 0.270 | 50 | 30 | 0.6 | 0.5 |
| ^10^ | Photo-lithography | Microcracked Au | No | Yes | 50 | 15 | 50 | 0.043 | 0.0009 | >1 (PDMS) |
| ^10^ | Photo-lithography | Microcracked Au | No | Yes | 25 | 21 | 25 | 0.043 | 0.0017 | >1 (PDMS) |
| ^10^ | Photo-lithography | Microcracked Au | No | Yes | 12 | 27 | 12 | 0.043 | 0.0036 | >1 (PDMS) |
| ^11^ | Photo-lithography | PEDOT:PSS | No | No | 10 | 980 | 5 | 2.5 | 0.50 | 0.03 |
| ^12^ | Laser engraving | PEDOT:PSS | Yes | Yes | 150 | 4.9 x 10^3^ | 150 | 2 | 0.013 | 0.25 |
| ^13^ | Thermal drawing | AgNWs | Yes | No | 200 | 21 | 105 | 1 | 0.01 | >4 |
| ^14^ | Extrusion | CNTs | Yes | No | 100 | 41 | N.R | N.R | N.R | 0.974 |
| ^15^ | Thermal evaporation | Microcracked Au | No | No | 150 | 15 | 150 | 0.035 | 0.00023 | >1 (PDMS) |
| ^16^ | Photo-lithography | Thin film Au | No | No | 120 | 16 | 120 | 0.035 | 0.00029 | >1 (PDMS) |
| ^17^ | Thermal evaporation | Thin film Au and PEDOT:PSS | No | No | 200 | 13 | 200 | 0.03 | 0.00015 | >1 (PDMS) |
| ^18^ | Thermal evaporation | CNTs | No | Yes | 500 | 10 | 500 | 2.9 | 0.01 | 1.3 (PDMS) |
| ^19^ | Moulding | Ag-AU core-sheath NWs | Yes | No | 500 | 0.010 | 500 | 60 | 0.12 | >1 (SBS) |
| ^20^ | Moulding | AgNWs | Yes | No | 500 | 0.100 | 500 | 10 | 0.02 | >1 (SBS) |
| ^21^ | Moulding | Ag Flakes | Yes | No | 500 | 3.027 | 500 | 100 | 0.20 | >1 (PDMS) |
| ^22^ | Photo-lithography | AgNW | No | Yes | 10 | 0.606 | 10 | 3 | 0.30 | >1 (PDMS) |
| ^22^ | Photo-lithography | AgNW | No | Yes | 20 | 0.706 | 20 | 3 | 0.15 | >1 (PDMS) |
| ^22^ | Photo-lithography | AgNW | No | Yes | 30 | 0.877 | 30 | 3 | 0.10 | >1 (PDMS) |
| ^23^ | Photo-lithography | PPy | No | No | 500 | 5.937 | 500 | 15 | 0.03 | >10 |
| ^24^ | Photo-lithography | PPy | No | No | 300 | 7.874 | 300 | 15 | 0.05 | >1 (PDMS) |
| ^25^ | Laser ablation | AgF and AgNPs | Yes | Yes | 200 | 0.003 | 200 | 200 | 1.00 | >1(SIS) |
| ^26^* | 3D printing | AgNPs | No | No | N.A | N.A | 3 | N.A | N.A | >1 (PU) |
|  | | | | | | | | | | |


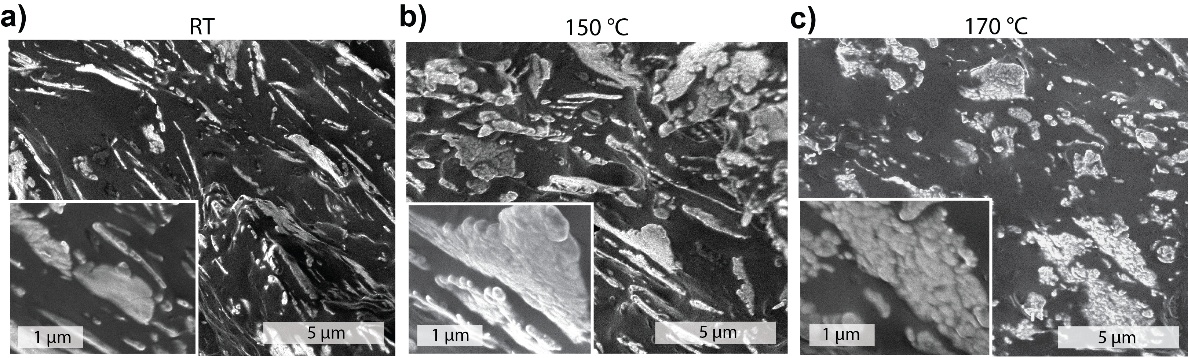


**Figure S1.** SEM images of liquid N_2_ cracked samples sintered at a) RT, b) 150°C, and c) 170°C.


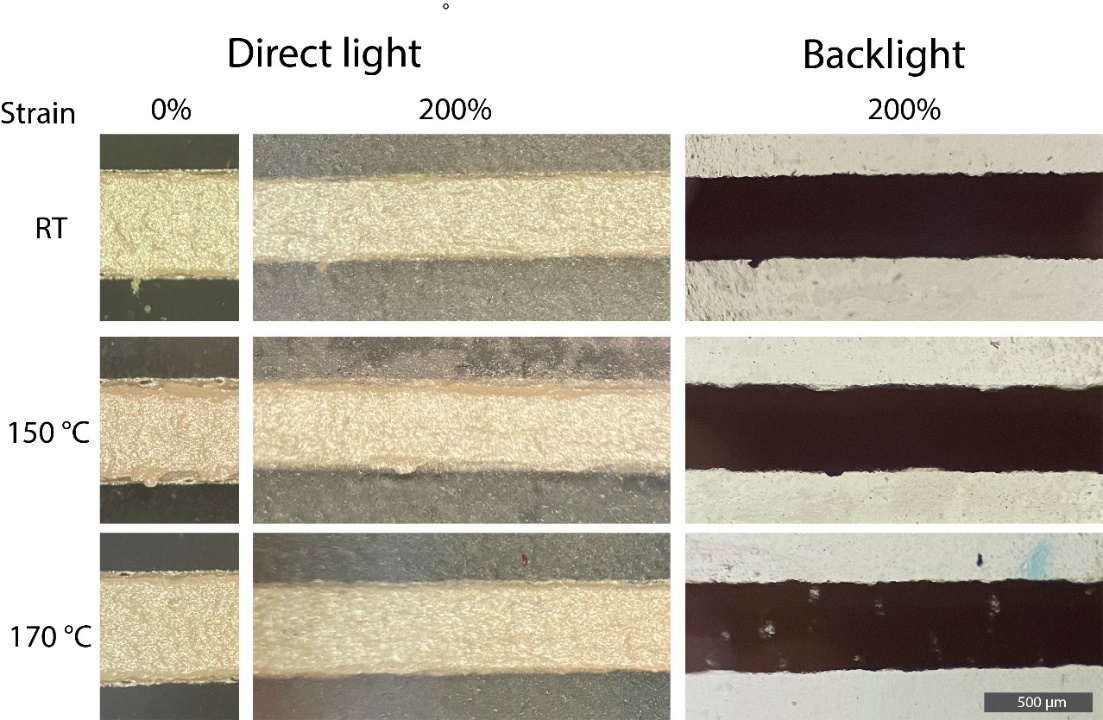


**Figure S2.** Direct light and backlight microscopy of original and deformed stretchable conductors sintered at different temperatures.

**
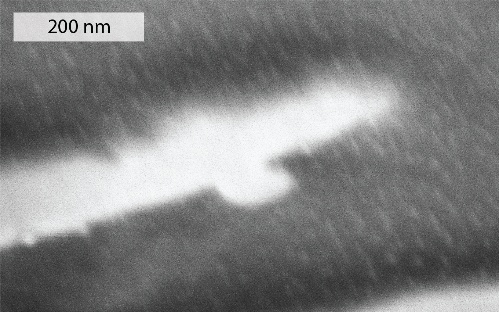
**

**Figure S3.** SEM imaging of the formed silver nanoparticles from AgF in the FIB samples.


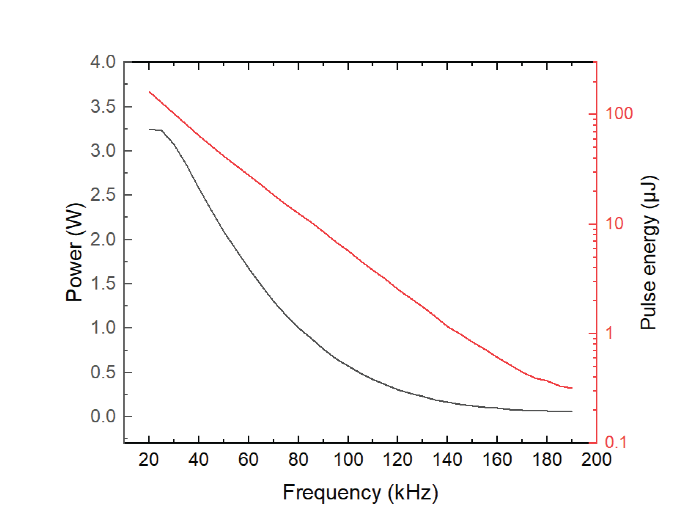


**Figure S4.** Measured output power and calculated pulse energy of the 355 nm UV laser system as a function of frequency.


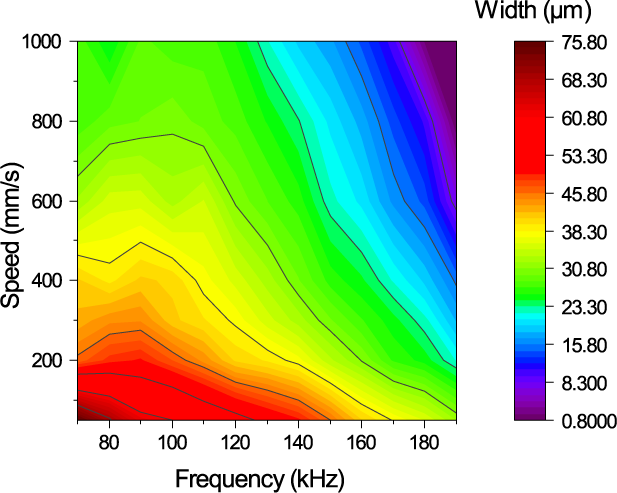


**Figure S5.** Width of the ablated lines for 355nm UV lasering at different frequencies and scanning speeds at one loop for 85 µm thick mask.


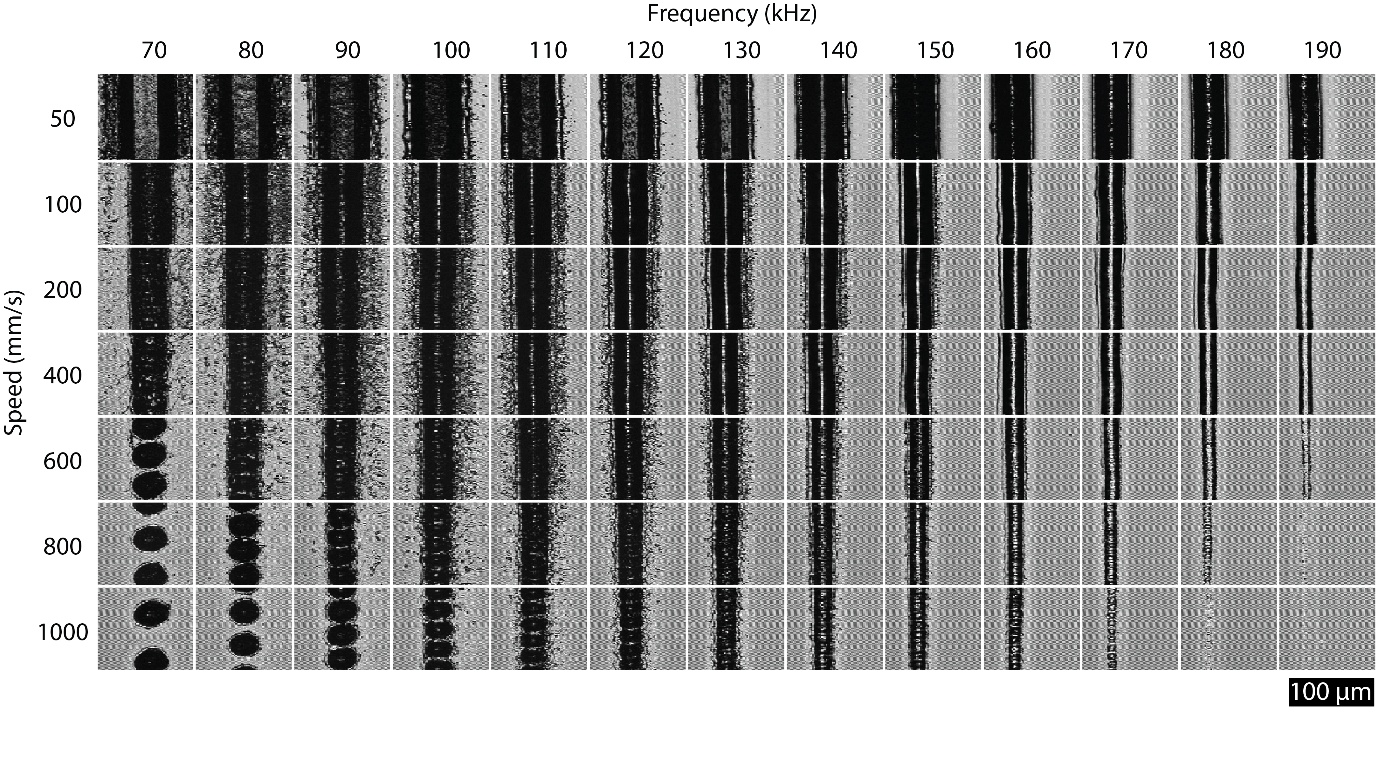


**Figure S6.** Grayscale optical image of the 355 nm UV laser ablation at different frequencies and scanning speeds at one loop for 85 µm thick mask.


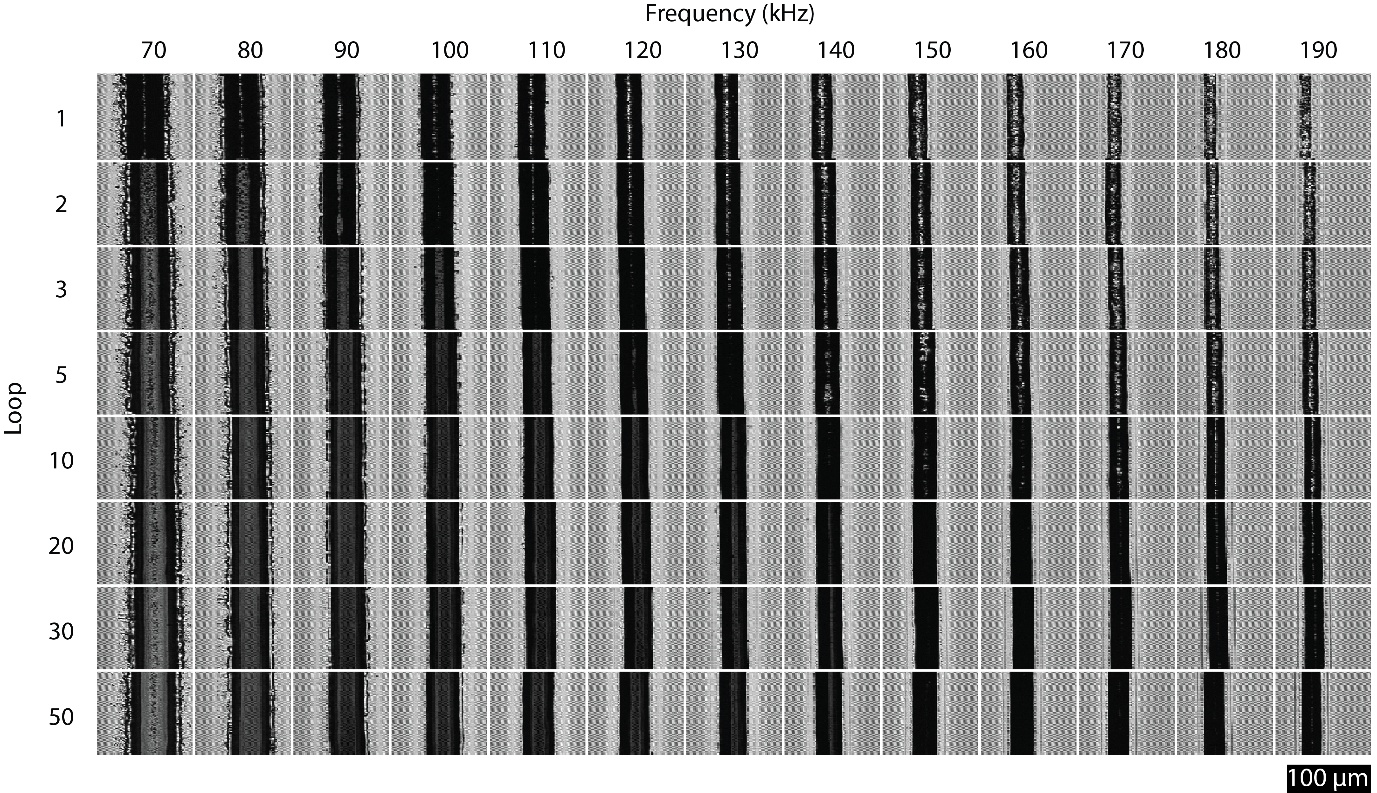


**Figure S7.** Grayscale optical image of the 355 nm UV laser ablation at different frequencies and loop counts at 100 mm/s speed for an 85 µm thick mask.


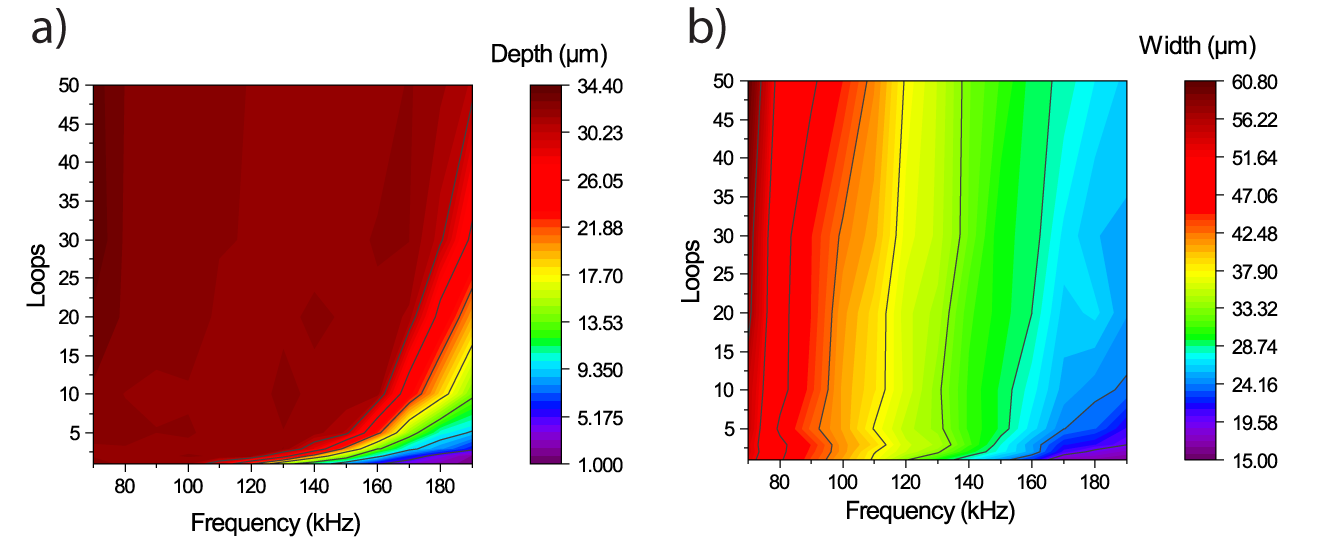


**Figure S8.** a) Depth and e) width of the 355 nm UV laser ablation at different frequencies and loop counts at 100 mm/s speed for a 35 µm thick mask.


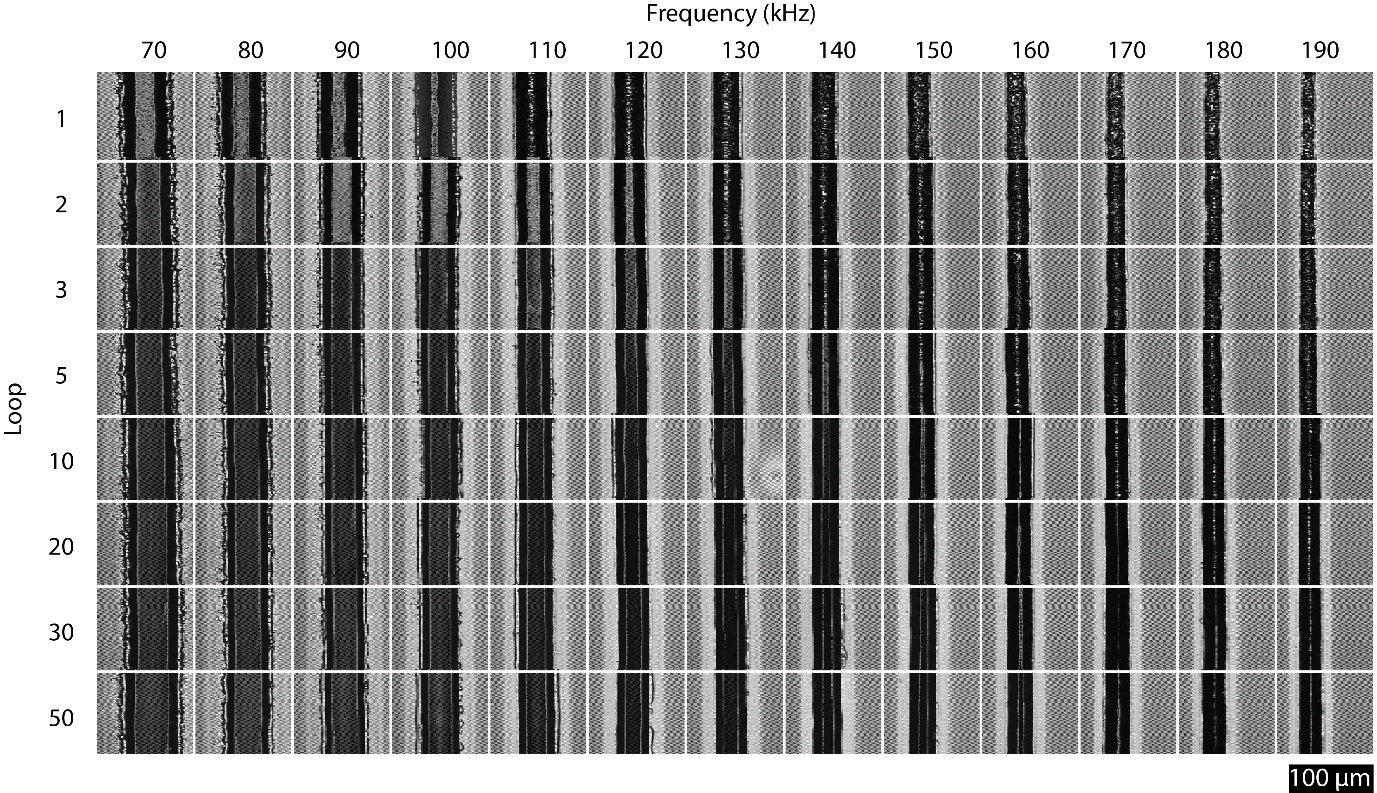


**Figure S9.** Grayscale optical image of the 355 nm UV laser ablation at different frequencies and loop counts at 100 mm/s speed for a 35 µm thick mask.


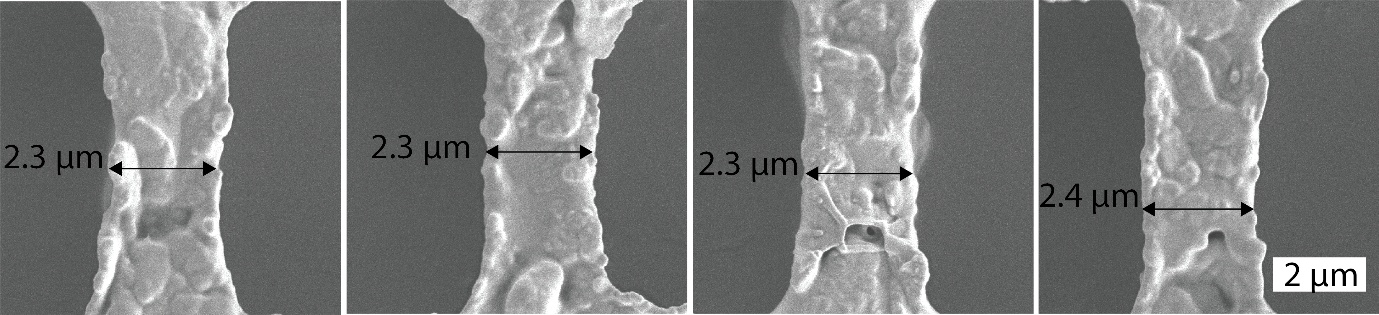


**Figure S10.** Printed stretchable conductor using a high resolution and high AR laser defined 3 µm thick mask and the measured widths.


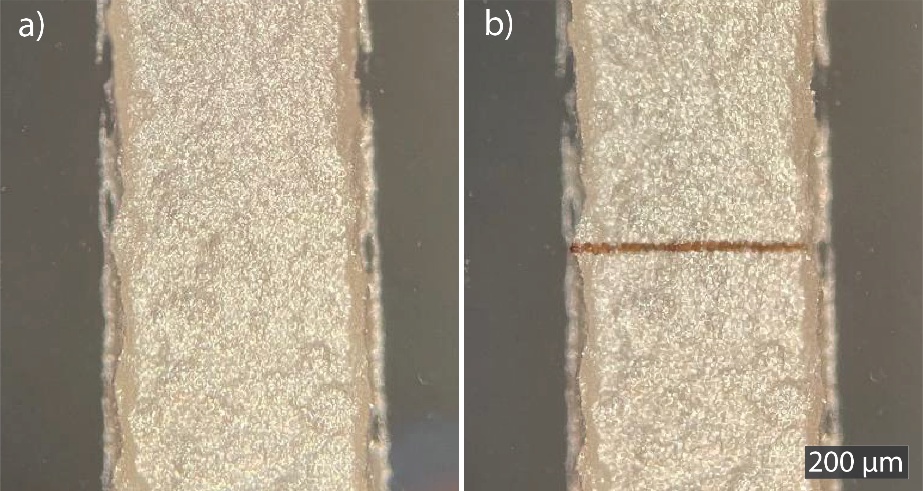


**Figure S11.** A stencil printed stretchable conductor encapsulated with 130 µm DS, before and after applying 355 nm UV laser (1 loops, 100 mm/s, 130 kHz).


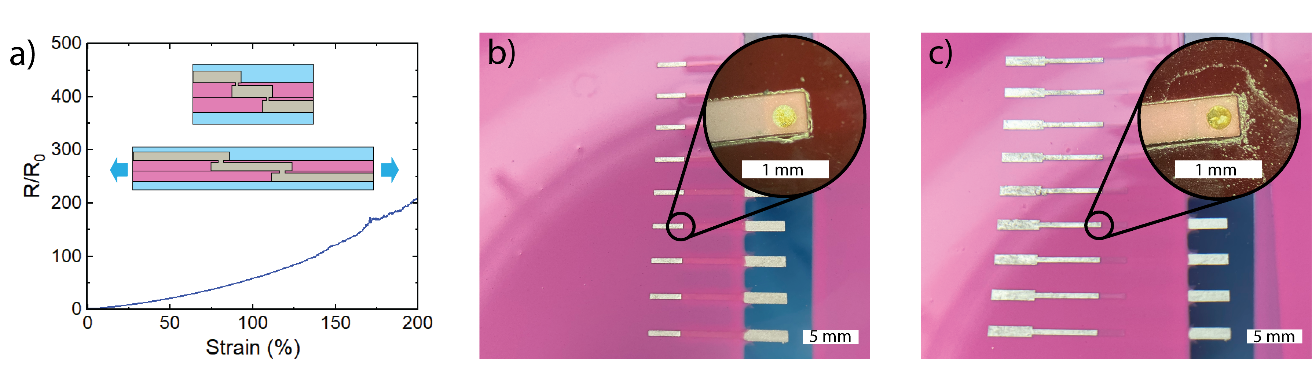


**Figure S12**. Multilayer printing of stretchable conductors. a) Electromechanical performance of a three-layer conductor connected via two 300 µm in diameter VIAs. b) Three-layer conductor sample after printing the second conductor layer, and c) after printing the third conductor layer (insets show microscope images of the selectively laser-ablated PVAL masks and drilled VIA hole connecting to the previous layers).

**
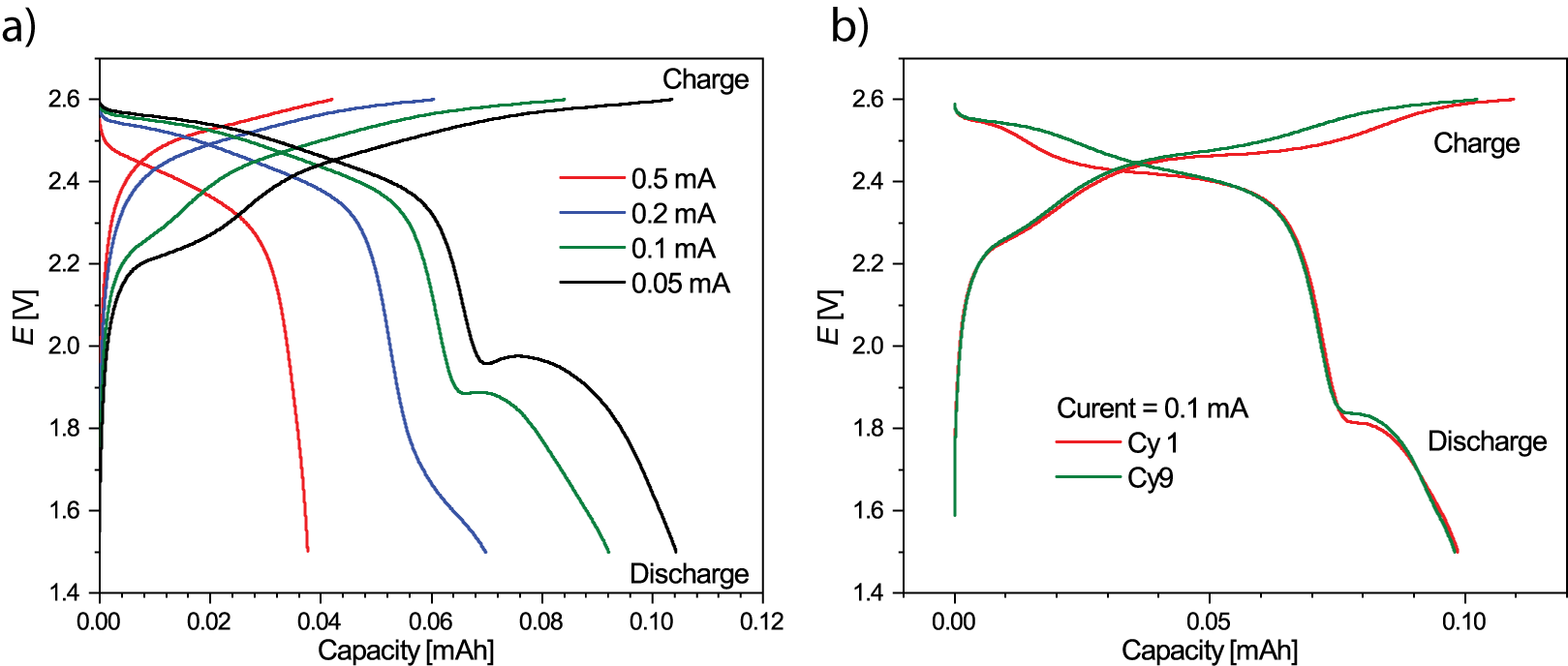
**

**Figure S13.** Micro battery electrochemical performance. a) Charge-discharge at different current rates. b) Stability test of the charge discharge performance over 9 cycles.

**
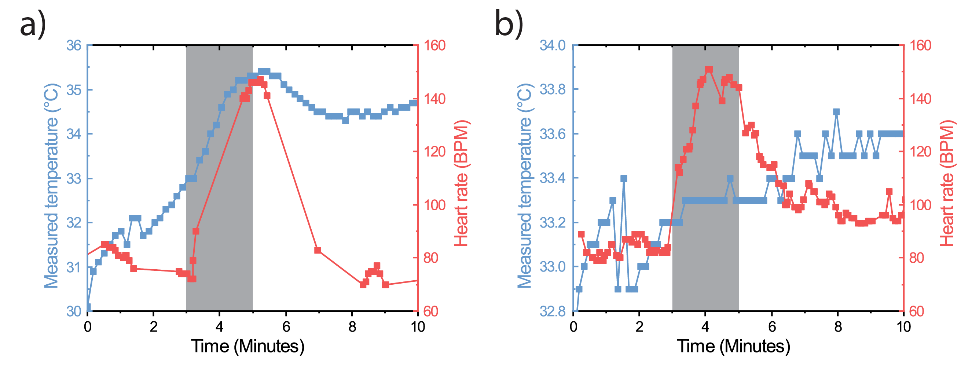
**

**Figure S14.** Measured body temperature using the soft temperature logger and heart rate before, during, and after a physical activity for two additional trials.


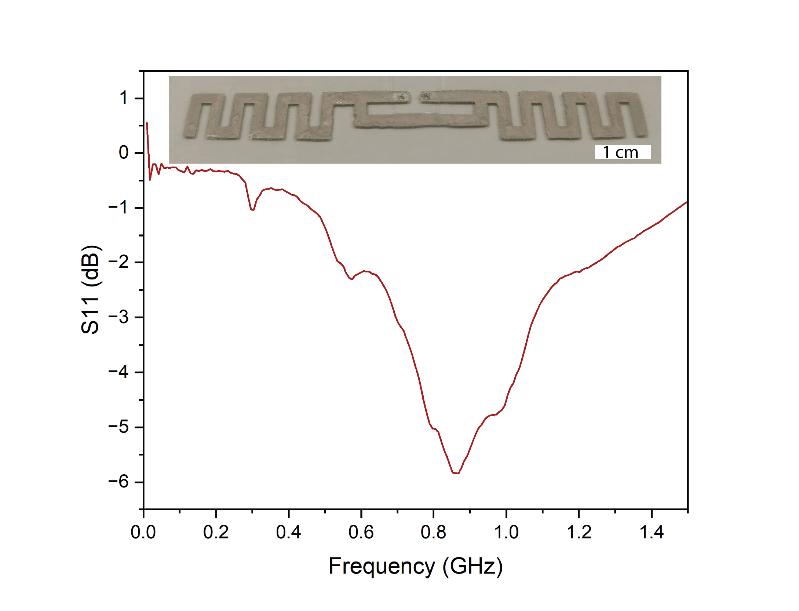


**Figure S15.** S11 parameter of the printed GHz-range antenna (shown in the inset) as a function of frequency.


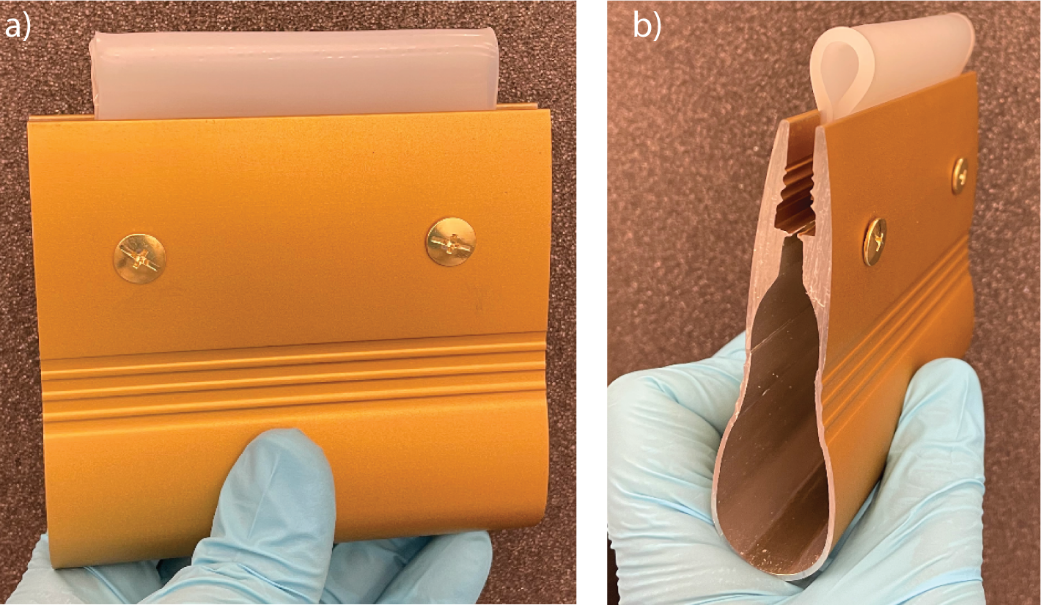


**Figure S16.** Soft squeegee. a) Front view and b) side view.

**References**

1 Martinez, V. et al. Stretchable Silver Nanowire–Elastomer Composite Microelectrodes with Tailored Electrical Properties. ACS Applied Materials & Interfaces 7, 13467-13475, doi:10.1021/acsami.5b02508 (2015).

2 Tybrandt, K. et al. High-Density Stretchable Electrode Grids for Chronic Neural Recording. Advanced Materials 30, 1706520, doi:10.1002/adma.201706520 (2018).

3 Jung, D. et al. Highly conductive and elastic nanomembrane for skin electronics. Science 373, 1022-1026, doi:doi:10.1126/science.abh4357 (2021).

4 Choi, S. et al. Stretchable Heater Using Ligand-Exchanged Silver Nanowire Nanocomposite for Wearable Articular Thermotherapy. ACS Nano 9, 6626-6633, doi:10.1021/acsnano.5b02790 (2015).

5 Araromi, O. A. et al. High-Resolution, Large-Area Fabrication of Compliant Electrodes via Laser Ablation for Robust, Stretchable Dielectric Elastomer Actuators and Sensors. ACS Applied Materials &amp; Interfaces 7, 18046-18053, doi:10.1021/acsami.5b04975 (2015).

6 Song, S. et al. Photothermal Lithography for Realizing a Stretchable Multilayer Electronic Circuit Using a Laser. ACS Nano 17, 21443-21454, doi:10.1021/acsnano.3c06207 (2023).

7 Lee, B. et al. Omnidirectional printing of elastic conductors for three-dimensional stretchable electronics. Nature Electronics 6, 307-318, doi:10.1038/s41928-023-00949-5 (2023).

8 Tybrandt, K. et al. Fast and Efficient Fabrication of Intrinsically Stretchable Multilayer Circuit Boards by Wax Pattern Assisted Filtration. Small 12, 180-184, doi:10.1002/smll.201502849 (2016).

9 Matsuhisa, N. et al. Printable elastic conductors with a high conductivity for electronic textile applications. Nature Communications 6, 7461, doi:10.1038/ncomms8461 (2015).

10 Adrega, T. et al. Stretchable gold conductors embedded in PDMS and patterned by photolithography: fabrication and electromechanical characterization. Journal of Micromechanics and Microengineering 20, 055025, doi:10.1088/0960-1317/20/5/055025 (2010).

11 Liu, Y. et al. Soft and elastic hydrogel-based microelectronics for localized low-voltage neuromodulation. Nature Biomedical Engineering 3, 58-68, doi:10.1038/s41551-018-0335-6 (2019).

12 Liu, Y. et al. Morphing electronics enable neuromodulation in growing tissue. Nature Biotechnology 38, 1031-1036, doi:10.1038/s41587-020-0495-2 (2020).

13 Lu, C. et al. Flexible and stretchable nanowire-coated fibers for optoelectronic probing of spinal cord circuits. Science Advances 3, e1600955, doi:10.1126/sciadv.1600955 (2017).

14 Zheng, X. et al. Soft Conducting Elastomer for Peripheral Nerve Interface. Advanced Healthcare Materials 8, 1801311, doi:10.1002/adhm.201801311 (2019).

15 Minev, I. R. et al. Electronic dura mater for long-term multimodal neural interfaces. Science 347, 159-163, doi:10.1126/science.1260318 (2015).

16 Musick, K. M. et al. Chronic multichannel neural recordings from soft regenerative microchannel electrodes during gait. Scientific Reports 5, 14363, doi:10.1038/srep14363 (2015).

17 Decataldo, F. et al. Stretchable Low Impedance Electrodes for Bioelectronic Recording from Small Peripheral Nerves. Scientific Reports 9, 10598, doi:10.1038/s41598-019-46967-2 (2019).

18 Yan, D. et al. Ultracompliant Carbon Nanotube Direct Bladder Device. Advanced Healthcare Materials 8, 1900477, doi:10.1002/adhm.201900477 (2019).

19 Choi, S. et al. Highly conductive, stretchable and biocompatible Ag–Au core–sheath nanowire composite for wearable and implantable bioelectronics. Nature Nanotechnology 13, 1048-1056, doi:10.1038/s41565-018-0226-8 (2018).

20 Park, J. et al. Electromechanical cardioplasty using a wrapped elasto-conductive epicardial mesh. Science Translational Medicine 8, 344ra386-344ra386, doi:10.1126/scitranslmed.aad8568 (2016).

21 Guvanasen, G. S. et al. A Stretchable Microneedle Electrode Array for Stimulating and Measuring Intramuscular Electromyographic Activity. IEEE Transactions on Neural Systems and Rehabilitation Engineering 25, 1440-1452, doi:10.1109/tnsre.2016.2629461 (2017).

22 Tybrandt, K. et al. Multilayer Patterning of High Resolution Intrinsically Stretchable Electronics. Scientific Reports 6, 25641, doi:10.1038/srep25641 (2016).

23 Guo, L. et al. Stretchable Polymeric Multielectrode Array for Conformal Neural Interfacing. Advanced Materials 26, 1427-1433, doi:10.1002/adma.201304140 (2014).

24 Qi, D. et al. Highly Stretchable, Compliant, Polymeric Microelectrode Arrays for In Vivo Electrophysiological Interfacing. Advanced Materials 29, 1702800, doi:10.1002/adma.201702800 (2017).

25 Vural, M. et al. Soft Electromagnetic Vibrotactile Actuators with Integrated Vibration Amplitude Sensing. ACS Applied Materials &amp; Interfaces 15, 30653-30662, doi:10.1021/acsami.3c05045 (2023).

26 Kang, Y. et al. High‐Resolution Printable and Elastomeric Conductors from Strain‐Adaptive Assemblies of Metallic Nanoparticles with Low Aspect Ratios. Small 16, 2004793, doi:10.1002/smll.202004793 (2020).
